# Diabetic Kinome Inhibitors—A New Opportunity for β-Cells Restoration

**DOI:** 10.3390/ijms22169083

**Published:** 2021-08-23

**Authors:** Barbara Pucelik, Agata Barzowska, Janusz M. Dąbrowski, Anna Czarna

**Affiliations:** 1 Malopolska Centre of Biotechnology, Jagiellonian University, Gronostajowa 7A, 30-387 Krakow, Poland; barbara.pucelik@uj.edu.pl (B.P.); agata.barzowska@doctoral.uj.edu.pl (A.B.); 2 Faculty of Chemistry, Jagiellonian University, Gronostajowa 2, 30-387 Krakow, Poland

**Keywords:** diabetic kinome, protein kinases, DYRK1A, diabetes, beta-cells

## Abstract

Diabetes, and several diseases related to diabetes, including cancer, cardiovascular diseases and neurological disorders, represent one of the major ongoing threats to human life, becoming a true pandemic of the 21st century. Current treatment strategies for diabetes mainly involve promoting β-cell differentiation, and one of the most widely studied targets for β-cell regeneration is DYRK1A kinase, a member of the DYRK family. DYRK1A has been characterized as a key regulator of cell growth, differentiation, and signal transduction in various organisms, while further roles and substrates are the subjects of extensive investigation. The targets of interest in this review are implicated in the regulation of β-cells through DYRK1A inhibition—through driving their transition from highly inefficient and death-prone populations into efficient and sufficient precursors of islet regeneration. Increasing evidence for the role of DYRK1A in diabetes progression and β-cell proliferation expands the potential for pharmaceutical applications of DYRK1A inhibitors. The variety of new compounds and binding modes, determined by crystal structure and in vitro studies, may lead to new strategies for diabetes treatment. This review provides recent insights into the initial self-activation of DYRK1A by tyrosine autophosphorylation. Moreover, the importance of developing novel DYRK1A inhibitors and their implications for the treatment of diabetes are thoroughly discussed. The evolving understanding of DYRK kinase structure and function and emerging high-throughput screening technologies have been described. As a final point of this work, we intend to promote the term “diabetic kinome” as part of scientific terminology to emphasize the role of the synergistic action of multiple kinases in governing the molecular processes that underlie this particular group of diseases.

## 1. Introduction

The diabetic kinome consists of protein kinases that control and regulate protein functions involved in diabetes. A range of experimental evidence indicates that pharmacological modulations of the diabetic kinome are inextricably linked to changes in metabolic homeostasis. Numerous cell types and signaling pathways in diabetes have been identified (i.e., PI3K-AKT/PKB, ERK/MAPK, growth factor, and hormone signaling pathways) [1,2,3]. Among others, the selected proteins’ kinase activity, i.e., hexokinase, pyruvate kinase M2 ketohexokinase isoform A, phosphoglycerate kinase 1, and nucleoside diphosphate kinase 1 and 2 (NME1/2), contribute to altered metabolic homeostasis [4,5].

Insulin regulates glucose homeostasis by modulating protein kinases’ activity in target tissues. The impairment of the kinome response to insulin leads to insulin resistance. Thus, many kinases, including (i) Jun N-terminal kinase (JNK), (ii) I kappa beta kinase (IKK), (iii) protein kinase C (PKC) theta, (iv) glycogen synthase kinase 3 (GSK3), (v) S6 kinase-1 (S6K1), and (vi) 5’AMP-activated protein kinase (AMPK), are critical factors that regulate the insulin-dependent processes. Moreover, many of them are also related to the pathogenesis of diabetes [6]. More recently, the role of dual-specificity tyrosine phosphorylation-regulated kinase A (DYRK1A) was identified in β-cells’ function. Due to the large amount of data related to mutations, overexpression, and dysregulation of protein kinases in the pathogenesis of many diseases, this family of enzymes has become one of the most important drug targets during the past 20 years [7,8]. A milestone was the FDA approval (in 2001) of the first kinase inhibitor, imatinib, also known under the trade name Gleevec® (Novartis, Basel, Switzerland). It is an oral chemotherapy drug used to treat leukemia and gastrointestinal stromal tumors whose mechanism of action involves potent inhibition of the constitutively active BCR-ABL fusion protein [7,8,9]. Imatinib is also being studied in phase II clinical trials to treat type I diabetes (T1D) (NCT01781975) [10]. This study aimed to investigate the possibility of short-term therapy with imatinib to induce tolerance and long-term remission of T1D [10]. The development of small-molecule kinase inhibitors has emerged as one of the most extensively pursued areas of drug discovery. In recent years, significant progress has been made in the battle against diabetes mellitus (DM) in understanding its biological mechanisms. However, despite a large amount of data, the search for diabetes-relevant kinases inhibitors with their binding modes and structural features is required. Especially, there are still unexplored gaps in the knowledge of how protein kinases from the DYRKs family affect apoptosis, cell cycle regulation, cellular proliferation, and insulin resistance in diabetes. DYRK1A has been confirmed as a regulator of regenerative pathways essential for proper pancreatic β-cells function in humans. Inhibitors of this kinase have been extensively studied to treat various types of diabetes [11,12]. Harmine and its derivatives are one of the most frequently studied—and still the most potent therapeutic of this group of compounds [13,14,15,16].

**Figure 1:**
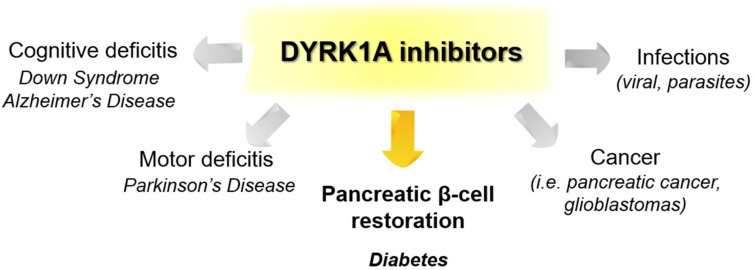
DYRK1A role in human diseases and the potential use of its inhibitors.

Recently published review papers on diabetes and CNS disorders highlight the importance of DYRK inhibitors in the therapy of cancer and neurological disorders [17,18,19] and suggest the directions in the design and development of small-molecule inhibitors (Figure 1) [12,20,21,22,23]. This review describes recent reports on the initial self-activation of DYRK1A by tyrosine autophosphorylation, the development of DYRK1A inhibitors, and their importance in the treatment of diabetes mellitus (DM). In addition, advances in understanding the structure and function of DYRK kinases and functions and emerging HTS technologies are described.

Modulating the activity of DYRK1A kinase, which is significantly involved in diabetes, with small-molecule inhibitors could be an attractive therapeutic strategy to tackle diabetes.

The global diabetes population is estimated to be 9.3% (463 million people) in 2019, rising to 10.2% (578 million) by 2030 and 10.9% (700 million) by 2045 [24]. Thus diabetes has become a challenging health problem affecting the global population, and the prevalence is higher in developing countries [24]. Diabetes mellitus (DM) is caused by chronic hyperglycemia due to impaired β-cells from the islets of Langerhans, distributed throughout the endocrine pancreas to produce appropriate insulin levels or ineffective insulin usage [25]. It is also associated with vascular complications, mainly diabetic neuropathy (DN), with an incidence of about 50% [26]. The DN progresses with decreasing nerve functionality with a high risk of pain, trophic changes, and autonomic dysfunction. Diabetes may also lead to ketoacidosis, retinopathy, nephropathy, and skin complications. Moreover, diabetes dramatically increases the risk of various cardiovascular problems, including coronary artery disease with chest pain (angina), heart attack, stroke, and narrowing of arteries (atherosclerosis) [27,28].

According to the latest classification, there are several types of diabetes: type 1 diabetes designated as T1D, type 2 diabetes marked as T2D, gestational diabetes, and other variants listed in Table 1. It should be emphasized that the pathogenesis of each form differs significantly. T1D is an autoimmune disorder caused by the T-cell-mediated destruction of the insulin-producing pancreatic β-cells. T2D is a consequence of impaired glucose tolerance and insulin resistance with the prominent risk factors: obesity and physical inactivity. In addition to these most common, there is also monogenic diabetes (for example, MODY, neonatal diabetes), extrinsic pancreatic diseases (for example, cystic fibrosis-related diabetes, pancreatic diabetes [or type 3c], and drug-induced diabetes [29]. In type 1 diabetes, a significant reduction in pancreatic β-cells, resulting in insulin insufficiency and hyperglycemia, is observed. Type 2 diabetes is associated with insulin resistance, which causes the compensatory expansion of pancreatic β-cells and increases plasma insulin levels [30,31]. Finally, insufficient β-cell mass and insulin secretion also cause mature onset diabetes of the young and gestational diabetes [32]. Therefore, modern antidiabetic therapies are based on increasing functional pancreatic β-cell mass. This review briefly discusses only the most common forms of diabetes. According to the American Diabetes Association (ADA) position statement, “Diagnosis and Classification of Diabetes Mellitus” (Table 1) provides a detailed classification of diabetes by etiology [33,34].

## 2. Insulin Homeostasis and Diabetes

**Figure 2:**
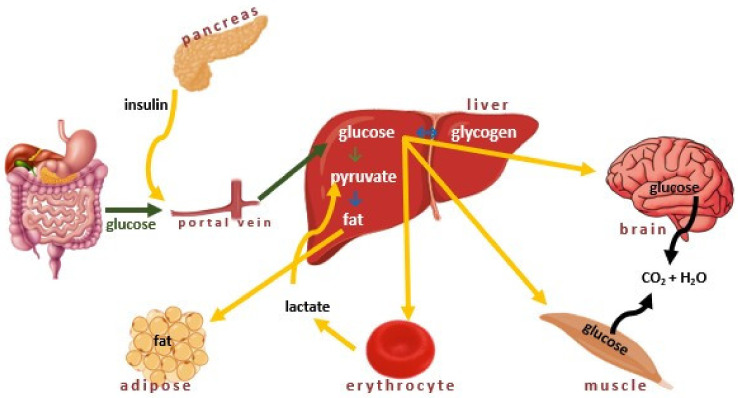
The flow chart illustrating the role of the pancreas in regulating blood glucose concentration. Localized in the islets of Langerhans, pancreatic β-cells respond to blood glucose levels, resulting in the release of the proper amounts of insulin. Insulin affects the liver, muscles, brain, erythrocytes, and adipocytes. The loss of β-cells leads to insufficient insulin production, resulting in increased blood glucose levels and eventually causes diabetes or insulin resistance. Adopted, modified, and re-drawn from [36].

Insulin plays a crucial role in many metabolic processes, including (i) facilitation of cellular uptake of glucose, (ii) prevention of the glucose release by the liver, (iii) activation of the muscle cells to take up amino acids, and (iv) reduction the breakdown, conversion, and release of fats (Figure 2) [35]. In several tissues, such as the liver, muscle, and adipose tissue, insulin participates in glucose metabolism by stimulating glucose uptake and influencing both glycolysis and gluconeogenesis.

**Figure 3:**
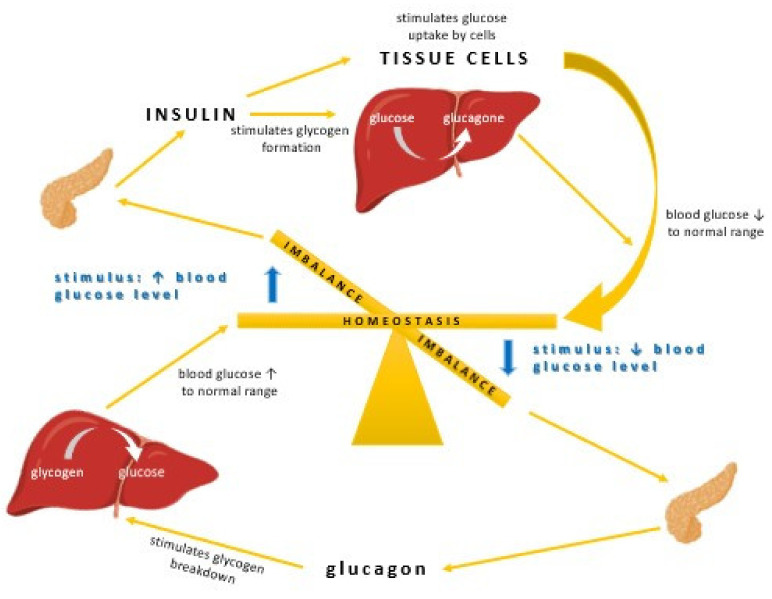
Scheme illustrating possible effects of insulin and glucagon on body function. Adopted, modified, and re-drawn from [41].

Insulin stimulates glycogen synthesis by inhibiting glycogen synthetase kinase, enhances its production through mTOR activation, promotes fatty acid synthesis, and inhibits lipolysis by activating Acetyl-CoA Carboxylase. It also inhibits hormone-sensitive lipase and modulates gene transcription through the MAPK pathway or Akt-mediated phosphorylation of FOXO transcription factors [37]. The secretion of insulin from the β-cells can be triggered either by somatotropin or by glucagon. The most important stimulant for insulin release is glucose, and when blood glucose levels rise, insulin is released to balance this process. The deficiency in insulin regulatory function may be caused by inadequate insulin secretion and/or reduced tissue response. It also results from a complete inability of islet cells to produce insulin (T1D) or the failure to produce enough insulin (Figure 3) [38,39,40].

Diabetes is a heterogeneous disease, but most cases corresponding to type 1 and type 2 diabetes. Nevertheless, a considerable proportion of patients does not fit into this classification and are known to have hyperglycemia caused by a mutation in a single gene. Despite the rapid evolution of molecular diagnosis methods, many MODY cases may be misdiagnosed as type 1 or type 2 diabetes. Thus, in the following sections, we briefly characterized only these most common types of diabetes.

### 2.1. Type I Diabetes (T1D)

T1D, referred to as insulin-dependent diabetes or juvenile-onset diabetes, concerns ca. 5–10% of cases. As already mentioned, it results from autoimmune destruction of the β-cells, accompanied by cellular invasion by both CD4+ and CD8+ T cells, leading to a decrease in β-cell mass [42,43]. Several markers of β-cell immune destruction include autoantibodies to islet cells, insulin, glutamic acid decarboxylase (GAD65), and tyrosine phosphatases IA-2 and IA-2β that are usually present in ca. 90% of patients [34]. T1D has genetic predispositions; the human leukocyte antigen (HLA) complex linked to the DQA and DQB genes constitutes the most relevant susceptibility region [44,45]. It is also related to poorly defined environmental factors. A small percentage of T1D patients (<10%) display no autoimmune response evidence and are categorized as type 1B diabetic or idiopathic population [43].

### 2.2. Type 2 Diabetes (T2D)

The most common form of diabetes is T2D (ca. 90–95% cases), also named non-insulin-dependent diabetes or adult-onset diabetes. Even though the etiologies of T2D are not fully explored. In this case (in contrast to T1D), autoimmune destruction of β-cells does not occur [25]. Lifestyle factors, including physical inactivity, sedentary lifestyle, smoking, and frequent alcohol consumption, play an important role in developing T2D [3,31]. It is characterized by increased hyperinsulinemia, insulin resistance, and β-cell dysfunction, with up to 50% cell loss at the time of diagnosis. T2D leads to a decrease in glucose transport into the liver, muscle cells, and fat cells. Recently, the involvement of impaired α-cell function has been recognized in the pathophysiology of T2D [46]. Consequently, glucagon and hepatic glucose levels that rise during fasting are not suppressed with a meal due to inadequate insulin concentration, increased insulin resistance, and increased fat breakdown with hyperglycemia [32]. 

**Figure 4:**
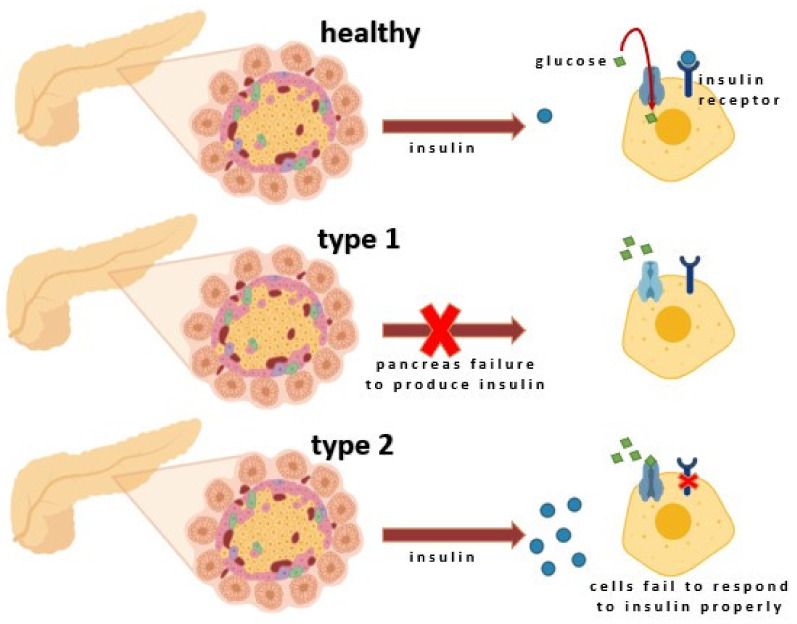
Schematic illustration of the main types of diabetes in which the pancreas does not produce enough insulin, or the body’s cells do not respond appropriately to the insulin produced. Adopted and changed from a stock image.

T2D contributes to a substantial increase in the risk of cardiovascular disease. Other mechanisms for developing hyperglycemia-induced micro and macrovascular complications include endothelial dysfunction, advanced glycation end-product formation, hypercoagulability, increased platelet reactivity, and overexpression of sodium-glucose co-transporter-2 (SGLT-2) [47,48,49]. Fibrinolysis and platelet aggregation can be remarkably improved by metformin therapy. Glucagon-like peptide-1 (GLP-1) receptor agonists have been confirmed to have protective effects on the endothelium, which may help to reduce inflammation (Figure 4.) [50,51,52].

### 2.3. Maturity-Onset Diabetes of the Young (MODY)

Other forms of diabetes are associated with monogenetic defects in β-cell function. In maturity-onset diabetes of the young (MODY), the onset of hyperglycemia occurs early (generally before age 25). This type of DM is characterized by impaired insulin secretion and minimal or no defects in insulin action [53]. The most common form of MODY is associated with mutations in the hepatic transcription nuclear factor HNF-1α [54,55,56,57]. It can also be related to mutations in the glucokinase gene, which serves as a “glucose sensor” for the β-cell [58,59]. Due to the impairments in the glucokinase gene, higher plasma glucose levels are required to elicit normal insulin secretion [60]. The critical factor distinguishing MODY from type 1 diabetes is the autoantibody negativity. Although GADA was reported in 1% of individuals with MODY, the undetectable C-peptide concentration and lower HbA1c (GCK-MODY) may be determined [61,62]. Besides the genetic background, the features distinguishing MODY diabetes from T2D are the onset of the disease usually in the second or third decade of life, most often the absence of obesity, lower BMI, and the predominance of insulin secretion defect in the absence of insulin resistance or even high insulin sensitivity [63].

### 2.4. Alzheimer’s Disease—Diabetes Mellitus (AD-DM) Relation and Type 3 Diabetes (T3D)

**Figure 5:**
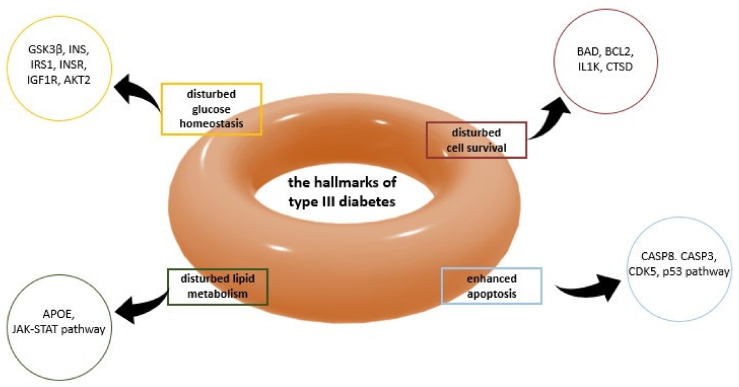
Diagram illustrating the hallmarks of type III diabetes. Adopted and modified from [80].

In recent years, a significant increase in the incidence of Alzheimer’s Disease related to T2D, is observed. Patients with T2D are almost twice as likely to develop AD than patients who only have insulin resistance. T2D and Alzheimer’s Disease patients have similar β-amyloid deposits in the pancreas as in the brain [64,65,66]. Several researchers have suggested that this new pathology is referred to as type 3 diabetes (T3D) (Figure 5) [67]. Some of the target receptors in T2D, e.g., IGF-1R, [68,69,70] PPAR, [71] IDE, [72,73], are also crucial regulators of tau protein expression and phosphorylation. For instance, it was reported that both hyperinsulinemia and IDE might be risk factors for Alzheimer’s disease [72,74]. 

The function of glucose transporter (GLUT) protein is controlled by the insulin-like growth factor (IGF) family, consisting of three ligands (insulin, IGF-1, and IGF-2), six IRs, and up to seven IGF-binding proteins (IGFBP1-7). IGF-1 and insulin can regulate neuronal excitability, metabolism, and survival through the insulin/IGF-1 signaling pathway. Few evidence on Alzheimer’s Disease patients’ brains showed a deficit ratio of insulin and resistance in IGF-1, suggesting that AD might be diabetes type 3 [74]. Nevertheless, several studies also suggest the protective role of insulin against apoptosis through various signaling pathways that suppress intracellular oxidative stress. For instance, the insulin/IGF/Akt pathway is considered to promote β-cell survival. [74].

The DYRK1A kinase is involved in molecular pathways relevant to human pancreatic β-cell proliferation, thereby providing a potential therapeutic target for β-cells regeneration in T1D and T2D [75,76]. A further target of DYRK1A has been identified as insulin receptor substrate-2 (IRS2) [77,78]. Moreover, hyperphosphorylation caused by DYRK1A overexpression has been implicated in many pathogenetic changes attributed to brain diseases, particularly in Down Syndrome and Alzheimer’s Disease [79,80]. 

## 3. Molecular Basis of Diabetes 

### 3.1. Diabetic Kinome

Protein kinases are key regulators of signal transduction pathways in many physiological processes. Protein phosphorylation catalyzed by them is one of the major intracellular mechanisms of structural and enzymatic protein regulation. Reversible phosphorylation/dephosphorylation is involved in all physiological events, and its disruption can lead to many pathological cases [81,82]. A kinome is a set of genes for protein kinases in its genome. Serine and threonine kinases contribute to insulin resistance and the development of diabetes (T2D) [83,84,85]. Kinases such as AMP-activated protein kinase (AMPK), Ikβ kinase (IKK), protein kinase C (PKC), and mitogen-activated protein kinases (MAPKs) play important roles in the development of insulin sensitivity and insulin resistance [6,86,87,88]. Rho-associated coiled-coil-containing protein kinase (ROCK) and RNA-activated protein kinase (PKR) are also involved in the pathogenesis of insulin resistance [89,90]. AMPK regulates lipid and glucose metabolism, therefore, this enzyme appears to be one of the main factors responsible for maintaining energy homeostasis in the body. Activation of this protein leads to inhibition of anabolic pathways, and its dysregulation is one of the mechanisms responsible for insulin resistance-induced diabetes [91,92]. Thus, understanding the interplay between diabetes and protein kinome may help develop the targeted drug therapies to minimize insulin resistance. Moreover, it may be critical for the prevention of diabetes. Therefore, the most crucial approach in discovering new drugs against diabetes is searching for pharmacological inhibitors of specific kinases. Initially, the focus was mainly on tyrosine kinase inhibitors and cancer indications, but the field is rapidly expanding towards serine/threonine kinases.

#### Inflammation, Diabetes, and Kinase Inhibition

Inflammation of pancreatic islets has emerged as a key contributor to the loss of functional β-cell mass in both T1D and T2D. In T1D, β-cells are the target of an autoimmune assault. Chronic low-grade inflammation and activation of the immune system are major factors in obesity-induced insulin resistance and T2D [93,94].

Obesity is a strong antecedent of T2D, and both diseases are associated with adverse cardiovascular risk profiles. Inflammatory pathways have been suggested as the underlying unifying pathogenic mediators for excess weight, diabetes mellitus, and cardiovascular diseases. Chronic inflammation is a common feature in the natural course of diabetes, and levels of inflammatory biomarkers (secreted mainly by adipocytes) correlate with prevalent and incident diabetes and major complications and cardiovascular diseases in particular [93,94]. The development of insulin resistance is also associated with low-grade tissue-specific inflammatory responses induced by various pro-inflammatory and/or oxidative stress mediators, notably pro-inflammatory cytokines such as IL-1β, IL-6, TNF-α, several chemokines, and adipocytokines. Chronic exposure of pro-inflammatory mediators stimulates cytokine-signaling proteins, which ultimately blocks insulin signaling receptors’ activation in β-cells of pancreatic islets [95].

Some of the protein kinases are directly involved in these inflammatory processes that underlie and accompany the progression of DM and its complications [95]. For instance, Iκβ kinase β (IKKβ), a central coordinator of inflammatory responses through activation of NF-κB, has been implicated as a critical molecular link between inflammation and metabolic disorders [96]. Phosphorylation by IKKβ targets IκBα to degrade proteasome that liberates NF-κB for translocation from the cytoplasm into the nucleus to promote expression of numerous target genes and consequently induce insulin resistance [74]. Xu et al. identified inhibitors of noncanonical Iκβ kinases (IKKs), TANK-binding kinase 1 (TBK1), and IκB kinase ε (IKKε), as enhancers of β-cell regeneration [97]. In the progression of T1D and T2D, a common feature is decreasing β-cell mass by cytokine- and/or glucolipotoxicity-induced apoptosis. Thus, prevention of β-cell loss by diabetic kinome inhibition can be an alternative approach for increasing β-cell mass in diabetes [97].

## 4. DYRK Family of Protein Kinases

Among the 518 human kinases, dual-specificity tyrosine phosphorylation-regulated kinase 1A (DYRK1A) is a conserved eukaryotic serine/threonine protein kinase. Other kinases belonging to the DYRKs family are DYRK1B, DYRK2, DYRK3, and DYRK4. DYRKs are from the CMGC group, which also includes other kinases: CDKs (cyclin-dependent kinases), CDKLs (CDK-like kinases), CK2 (casein kinase 2), CLKs (CDC-like kinases), GSKs (glycogen synthase kinases), and MAPKs (mitogen-activated protein kinases). Among them, CDKs, CKs, and MAPK have been well investigated in their functions in transcription, DNA damage repair, protein degradation, and neurogenesis [98,99]. However, DYRKs and CLKs in the signaling pathways remain not completely understood. DYRKs isoforms are subdivided into two classes based on their subcellular localization. DYRK1A/B belonging to class 1 are found in nuclei, whereas those of class 2 prefer cytoplasmic localization. They all possess a kinase domain [100]. 

### 4.1. DYRKs Activity and Regulation

**Figure 6:**
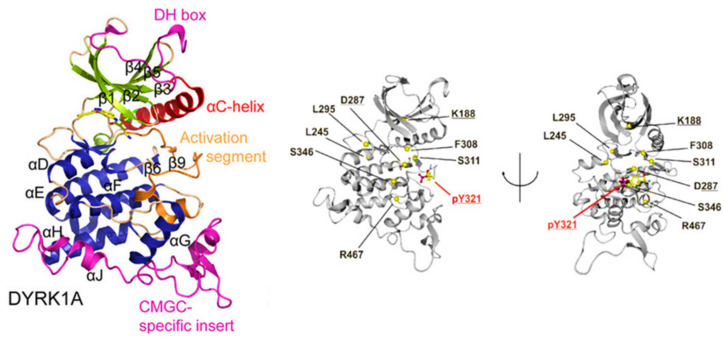
The DYRK1A kinase domain and DYRK homology box with the inhibitor DJM2005 bound in the ATP binding site. Magenta—the DH box and CMGC, orange—the activation segment (**left**). Ribbon representation of the kinase domain, with one orientation, rotated 90° around the depicted axis relative to the other (PDB entry 2VX3) (**right**) [104].

Many protein kinases can adopt an active and inactive conformation. The transition between these conformations is regulated by the reversible phosphorylation of discrete serine, threonine, or tyrosine residues in the ‘activation loop’ [101]. The DYRKs activation is dependent on the phosphorylation of a conserved tyrosine residue in the activation loop. The phosphorylated tyrosine forms salt bridges with two arginines in the P + 1 loop [102,103]. In DYRK1A, pY321 is important in the same interactions with two arginines (R325, R328) (Figure 6) [98,99,102,103,104].

DYRK possesses dual specificity, as it can autophosphorylate tyrosine Y321 in the activation loop and phosphorylate its substrates on either serine or threonine residues [103,105]. While dual MAPK phosphorylation is the primary process of upstream kinase regulation, tyrosine phosphorylation of DYRK and GSK3 occurs via autophosphorylation [106]. Activated DYRKs phosphorylate their substrates only on serine or threonine residues and cannot rephosphorylate on tyrosine. Therefore, a translational intermediate folding of DYRKs with different biochemical properties and tyrosine phosphorylation ability has been proposed [107]. It has also been suggested that the dual specificity of DYRKs is associated with dual sensitivity to kinase inhibitors [108]. Tyrosine phosphorylation during DYRKs activation is required to switch the conformation, but it does not maintain this state. For this purpose, the stabilizing effect of salt bridges formed between phosphotyrosine and the two arginines in the P + 1 loop may play a crucial role [102]. 

### 4.2. DYRK1A Expression and Its Role in Neurological Diseases

DYRK1A is a dosage-sensitive gene, and the imbalance in its expression affects brain structure and function [109]. It was reported that DYRK1A deficiency might lead to autosomal dominant mental retardation [109]. Therefore, both low and high DYRK1A expression can participate in developing several disorders [110]. DYRK1A expression is regulated by transcriptional factors, tumor suppressors, neurogenic factors, and protein-protein interactions. Reduced expression of the repressor complex AP4 results in premature overexpression of DYRK1A in the fetal brain [111]. It was also shown that the β-amyloid peptide increases DYRK1A mRNA SH-SY5Y cells [112]. 

Overexpression of another transcription factor, E2F1, enhanced DYRK1A activity by increasing its mRNA level in phoenix cells. Thus, DYRK1A may also be involved in cell-cycle regulation [113,114]. The DYRKs are key proteins regulating NFAT1 phosphorylation [115,116]. Overdosage of DYRK1A associated with the DSCR1 gene (resident of the “Down syndrome candidate region” and as a shock or stress gene) was reported to diminish NFATc activity in the immune response [117]. Protein p53‚ a 345 well-known tumor suppressor gene—has been identified to reduce DYRK1A expression. This process is mediated through the induction of miR-1246, resulting in the nuclear retention of NFATc1 and induction of apoptosis (overexpression of miR-1246 reduces DYRK1A levels) [118]. There is also evidence that upregulation of DYRK1A leads to changes in neuronal proliferation in Down Syndrome [119].

The WDR68 protein (also called HAN11, DCAF7) may act as a regulatory subunit of DYRK1A and DYRK1B [120,121]. Its overexpression inhibited the DYRK1A stimulation of GLI1-dependent reporter gene activity [122]. The circadian changes in DYRK1A levels have been reported, and DYRK1A was identified as a molecular clock component leading to CRY2 degradation [123]. Recently, SPRED1 and SPRED2 (sprouty-related protein with an EVH1 domain) were found to interact with the catalytic domain of DYRK1A, leading to the inhibition of phosphorylation of tau and STAT3 [124]. Thus, the DYRK1A-STAT pathway is involved in DS development. Phosphorylation by DYRK1A at tau Thr212 residue primes tau phosphorylation by GSK3 at the Ser208 residue, resulting in increased neurofibrillary accumulation tangles exists in the brain of Alzheimer’s Disease patients [125,126].

**Figure 7:**
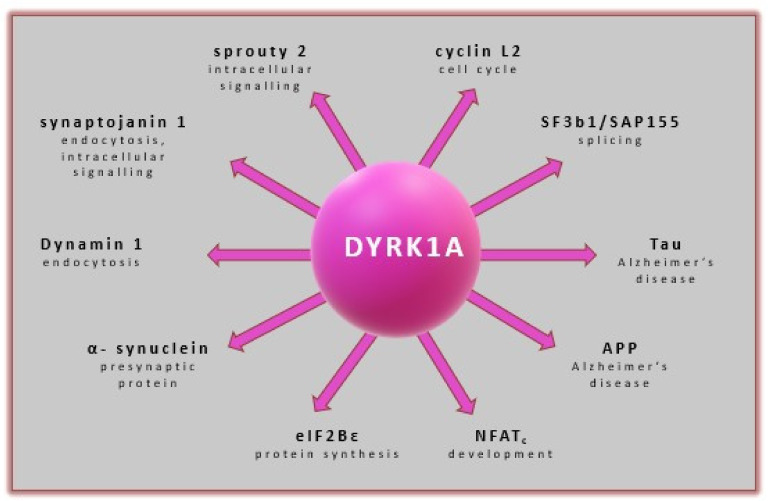
DYRK1A phosphorylation targets. Dual specificity tyrosine-phosphorylation-regulated kinase 1A (DYRK1A) is encoded by Hsa21 and phosphorylates multiple targets that play roles in various biological processes. Adopted and modified from [127].

DYRK1A plays an important role in cytoplasm homeostasis by localizing in the nucleus, as evidenced by increased immunoreactivity in this area [125]. The importance of DYRK1A in several biological processes is summarized in (Figure 7). 

### 4.3. DYRK1A Expression Affects Mechanisms of Diabetes 

**Figure 8:**
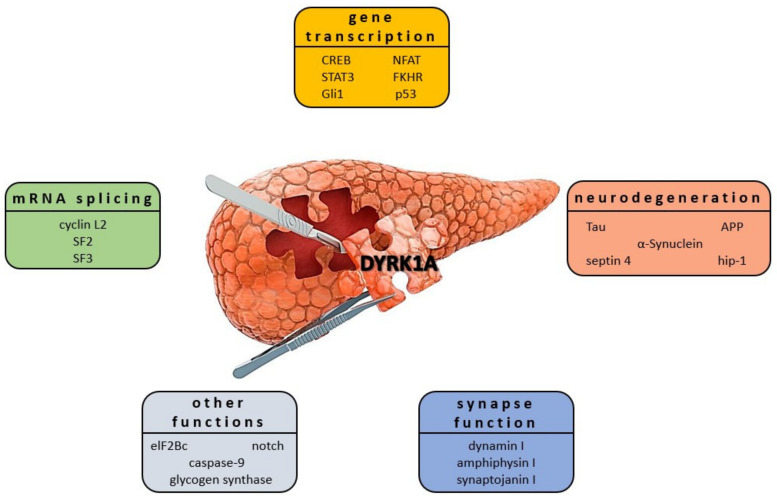
Scheme illustrating DYRK1A substrates and predicted roles in biological processes. Adopted and modified from [139].

DYRK1A has been found to affect multiple signaling processes in DM context by activating/inactivating signals of transcriptional and translation factors (RNAPII CTD [128], Sprouty2 [129], DREAM complex [130], CREB [131], FKHR [132]), splicing factors (regulation of Cyclin D1 turnover as well as miscellaneous proteins including caspase-9 [109,133,134], Notch [135], as well as glycogen synthase. It was shown that DYRK1A is involved in GSK3 phosphorylation at the Ser640 residue [136]. This interaction subsequently causes the activation of glycogen synthase, a key enzyme in glycogen synthesis regulated by insulin (Figure 8) [136,137]. DYRK1A is an important kinase for β-cell growth [138]. Studies using DYRK1A haploinsufficient mice have confirmed that they are burdened with severe glucose intolerance, reduced β-cell mass, and proliferation, leading to diabetes. Upregulation of DYRK1A in β-cells was found to enhance this phenomenon significantly [11,30]. DYRK1A emerged in the drug discovery field as one of the most attractive therapeutic targets for developing selective inhibitors as new drugs. They may have a high therapeutic potential for diabetes. The involvement of DYRK1A in the molecular pathways of different diseases is well described (see above). Therefore, we have focused on the new DYRK1A inhibitors discovered or specifically developed to provide the basis for the future development of these promising drugs.

## 5. Current Treatments of Diabetes

Pharmacological treatment of DM is based on the following strategies: (i) insulin infusion; (ii) administration of drugs that increase insulin secretion (sulfonylureas, meglitinides); (iii) enhancement of insulin sensitivity (metformin, thiazolidinediones); (iv) prevention of glucagon synthesis (DPP-4 inhibitors and GLP-1 receptor antagonists; and (v) application of substances that increase glucose excretion (SGLT-2 inhibitors) [140]. These strategies offer reasonable control of disease symptoms. However, there is simply no therapy to help DM patients to return to euglycemia. Thus, the focus on DM treatment’s development shifted toward population of functional, insulin-producing β-cells. It allows the patient to achieve insulin homeostasis and relieve hyperglycemia. One of the most promising advances in diabetes therapy is enabling β-cells to replicate. Several classes of drugs, hormones, or growth factors such as PPARg agonists, GLP-1 agonists, DPP-4 inhibitors, GSK3β inhibitors, prolactin, IGF-1, HGF, and PTHRP have been tested for their ability to stimulate β-cell proliferation [141,142]. However, all the proposed approaches failed in inducing β-cell proliferation in clinical conditions. Nevertheless, in the majority of treated diabetic patients, some β-cells were able to survive after the treatments listed above. In view of these considerations, it seems reasonable that modifications of current drugs or new appropriately designed small molecules or molecular targets could lead to β-cells proliferation/restoration. Under physiological conditions, human β-cells replicate at a low rate, about 2% per day, and only in the first few years of life. So far, attempts to stimulate adult human β-cells to replicate have failed pharmacologically. Nevertheless, it changed in 2015 with the discovery of harmine and other DYRK1A inhibitors, discussed below [16].

Harmine, a well-known DYRK1A kinase inhibitor, was identified by Wang et al., through a high throughput screening (HTS) campaign. It induces a mild level of c-Myc protein expression in rodent islets. The mechanism of action involves inhibiting the DYRK1A kinase (likely a primary target of harmine), which allows the NFAT pathway to induce c-Myc expression [16]. Moreover, it serves as the terminator of NFAT dephosphorylation by rephosphorylating NFAT and acts as a brake on the cell cycle [142]. Several DYRK1A inhibitors were identified among other known protein kinases inhibitors, and a few are used as tool compounds for β-cell regeneration [138]. 

### 5.1. DYRK1A Inhibitors for β-Cell Function Restoration

#### 5.1.1. Harmine and Its Analogues—SAR Approach

**Figure 9:**
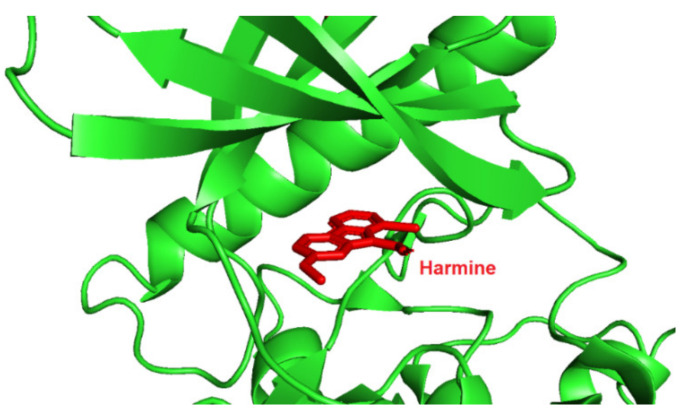
DYRK1A/harmine complex. DYRK1A ATP-binding pocket with harmine (PDB: 4YU2).

Screening of a panel of 69 kinases identified harmine as a potent DYRK1A inhibitor [143]. Comparative in vitro assays revealed that harmine is moderately specific towards DYRK1A [144]. The IC50 for DYRK1A has reached 33 nM. The DYRK1B showed an IC50 of 166 nM, and the other distant members DYRK2, DYRK3, and DYRK4 indicate IC50 values as following: 1.9 µM, 0.8 µM, and 80 µM [144]. Additionally, cell culture assays confirmed the potency of harmine for DYRK1A and its lack of toxicity. The DYRK1A ⁄ harmine complex’s crystal structure showed harmine blocking the ATP-binding pocket and interacting with the backbone NH of methionine on position 240 of the hinge-region, as well as with the conserved lysine on position 188, by forming two hydrogen bonds (Figure 9) [145]. Furthermore, the DYRK1A/harmine complex structure suggests that the accessible volume of the ATP-binding pocket can accommodate substituents at the β-carboline structure [102]. Consequently, harmine can likely be modified into an even more potent and selective DYRK1A inhibitor.

**Figure 10:**
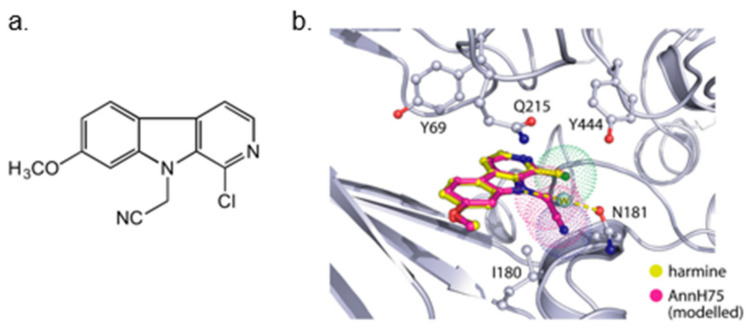
(**a**) Structure of AnnH75, (**b**) AnnH75 (magenta) docked into the harmine (yellow)-MAO-A structure (PDB 2ZGX). Adapted and modified from [146].

Although several new DYRK1A inhibitors have been identified and described so far, none meet the selectivity standards required for kinase-targeted probe molecules. Harmine has been recognized as a potent monoamine oxidase (MAO) inhibitor, which is associated with a number of side effects. Due to the limited selectivity of harmine, its derivative AnnH75 (Figure 10) was developed, which, unlike harmine, does not interact with MAO while maintaining DYRK1A inhibition. Epigallocatechin gallate (EGCG) from green tea has also been shown to be a DYRK1A inhibitor. 

**Figure 11:**
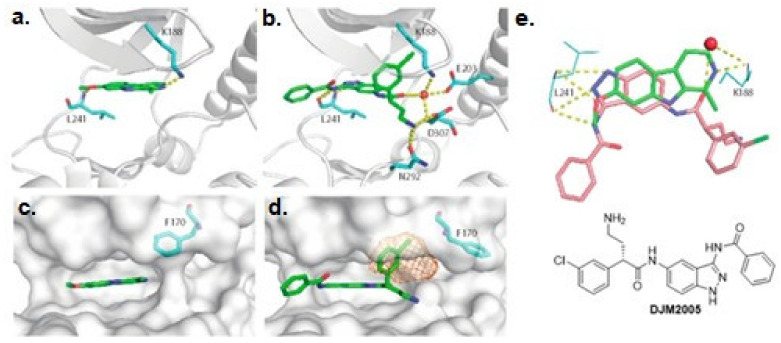
Comparison of harmine and DJM2005 binding to DYRK1A. (**a**) Harmine binds in the ATP-binding pocket of DYRK1A (PDB 3ANR). (**b**) DJM2005 interacts with the backbone of Leu241 and makes water-mediated contacts with the side chains Lys188, Glu203, and the backbone of Asp307. The primary amino group interacts with the side chain of Asn292 and Asp307 (PDB 2WO6). (**c**,**d**)The movement of Phe 170 in the DJM2005 complex creates a pocket (orange mesh) with DJM2005. (**e**) Superposition of the harmine (green) and DJM2005 (pink) complexes. Adapted and modified from [24].

In 2018 an integrated approach to investigate the structure-activity relationship of harmine derivatives for diabetes management (DYRK1A activity and β-cell proliferation) was developed [15]. Structure-based drug design and development were used to identify kinome and CNS off-targets and harmine-like molecules for more specific therapy. The crystal structure of DYRK1A with ATP-binding inhibitor DJM2005, 1, 7, and 9-amino harmine analogs were synthesized and examined in terms of their effect on DYRK1A binding and β-cell proliferation (Figure 11andFigure 12) [15,147,148].

**Figure 12:**
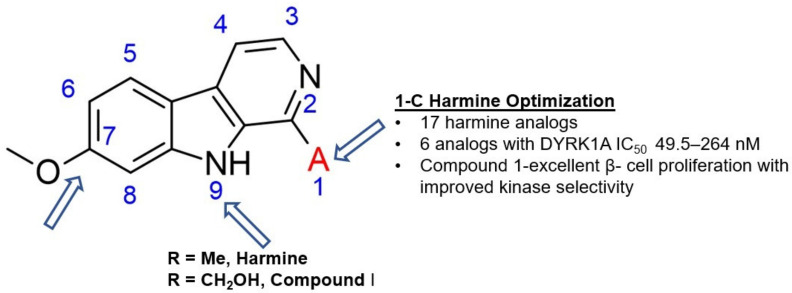
Harmine analogs.

Harmine analogs with polar substituents, e.g., hydroxymethyl or -acetyl, at position 1-C (Figure 12), showed good DYRK1A inhibition (IC50 49-67 nM). However, the 1-hydroxy moiety negatively impacted DYRK1A inhibition. The presence of a halogen atom at the position 1-C significantly increased the inhibition potency, making the 1-chloro substituted analog the most potent DYRK1A inhibitor, with an IC50 of 8.8 nM. Among synthesized 8 compounds with IC50 < 250 nM against DYRK1A, only four affected human β-cell proliferation. In contrast, the 1-amino analogs indicated no effect on β-cell proliferation. Notably, 1- and 3-hydroxymethyl compounds were most effective in vitro, indicating that these modifications improve the β-cell proliferation and potentially increase the selectivity toward DYRK1A [15].

In the subsequent paper, the same authors reported the set of harmine derivatives modified in 7-position as DYRK1A inhibitors with activity on human β-cell proliferation and targeted drug delivery [147]. The harmine backbone was substituted by terminal methyl ester, carboxamide, carboxylic acid, and amino/substituted amino groups with various carbon (1-5) chain lengths. Biochemical assays allow the selection of two 7-O analogs with activity toward DYRK1A (>100 nM). These compounds indicated an increase in β-cell proliferation (3-fold less than harmine). The reduced (in comparison to harmine) efficacy of described 7-O derivatives may be caused by several structural and biological factors, including lower potency for DYRK1A inhibition, limited cell permeability, reduced DYRK1A targeting and/or off-target kinase activity [147].

**Figure 13:**
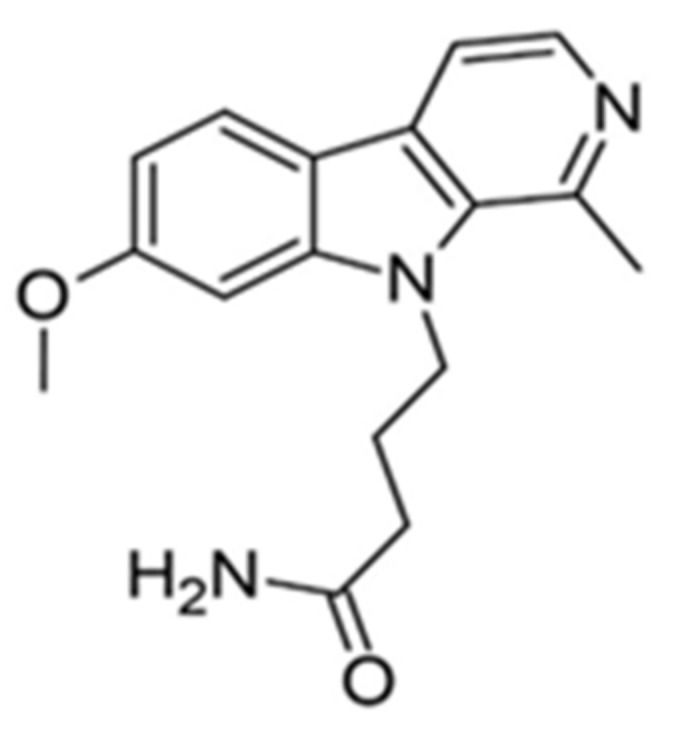
Chemical structure of 4-(7-methoxy-1-methyl-β-carbolin-9-yl)butanamide.

9-N-substituted analogues of harmine were also studied in order to eliminate the disadvantages of kinase and off-target [148]. The library of 62 compounds was tested, and among them, 4-(7-methoxy-1-methyl-β-carbolin-9-yl)butanamide proved to be the most promising DYRK1A inhibitor. The compound was tested in vivo and was significantly more effective than bare harmine (Figure 13). After treatment with 4-(7-methoxy-1-methyl-β-carbolin-9-yl)butanamide, Ki67 expression was increased in C57 mouse and human β-cells (Figure 13). In the PPX model, faster regeneration of β-cells was observed at a 10-fold lower drug dose than harmine. Similar results were also obtained in the NOD-SCID mouse model with transplanted human islets. Furthermore, no CNS side effects were observed at the dose of 30 mg/kg. Thus, this compound was selected as the lead candidate with high in vivo efficacy. Noteworthily identified inhibitor is also characterized by improved selectivity and CNS off-targets and superior activity in β-cells restoration—crucial for the treatment of diabetes [148]. The studies described above, the success of which has been confirmed in several animal models, demonstrate the validity of the modifications used within the harmine structure. This research direction is definitely worth continuing, but large-scale clinical trials will only provide answers to whether the most effective compound of this family will be equally effective in clinical treatment. 

#### 5.1.2. Perha Pharmaceutics Inhibitors

**Figure 14:**
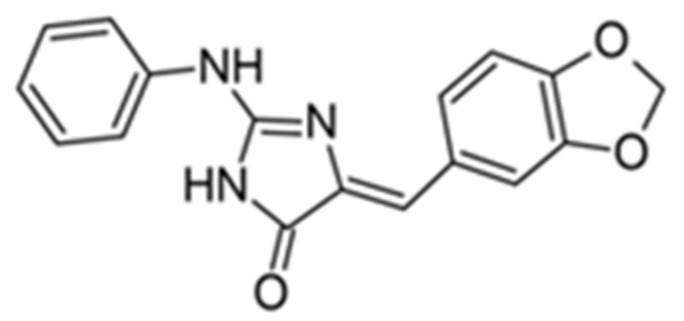
Chemical structure of L41, a compound derived from the marine sponge.

Despite the therapeutic potential of DYRK1A inhibitors, only a few of them have been well-characterized to date in terms of selectivity and biological effects [149]. These include a series of (i) pyrazolidine-3,5-dione derivatives, (ii) 6-arylquinazolin-5-amines, (iii) the β-carboline alkaloid harmine, (iv) the green tea polyphenol epigallocatechin-3-gallate, (v) the benzothiazole INDY, (vi) bauerine C derivatives, and (vii) leucettines [150]— a group of aminoimidazolinones derived from the marine sponge natural product leucettamine L41 was shown to be a most promising kinase inhibitor (Figure 14). The molecular interactions of leucettine L41 with its targets and its neuroprotective properties were extensively studied. [150] leucettine L41 (an ATP-competitive inhibitor of DYRKs and CLKs) may also interact with GSK3β and CK2. Moreover, it causes cellular effects, including pre-mRNA splicing, HT22 hippocampal cells protection cell death. Furthermore, it may induce autophagy and inhibit tau phosphorylation [151]. It was recently reported that leucettine L41 could prevent DYRK1A proteolysis, inhibit STAT3α phosphorylation, and reduce pro-inflammatory cytokine secretion (IL1-β, TNF-α, and IL-12) in APP/PS1 mice model. These results confirm the role of DYRK1A proteolysis in Alzheimer’s disease (AD) and suggest a possible mechanism as a novel target to counteract the disease [152].

**Figure 15:**
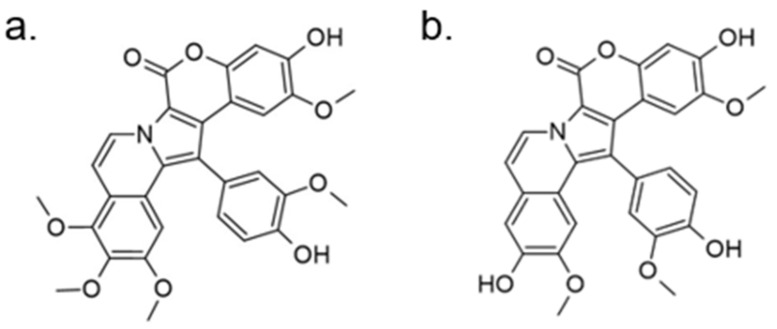
Structure of (**a**) lamellarin B and (**b**) lamellarin D.

Another approach for discovering DYRK1A inhibitors was screening a library of plant and fungal extracts. [153]. Several compounds were identified, including harmine, anthraquinone emodine and several flavonoids. These molecules were isolated and characterized as the active constituents from four plant extracts. However, due to the moderate activity of selected anthraquinone and flavonoids, the potential for further development is limited. In particular, flavonoids are known to be very promiscuous kinase inhibitors [153]. Lamellarins are natural marine products isolated from mollusks, ascidians, and sponges. Lamellarin D displays broad-spectrum kinase inhibition (i.e., CDK1, CK5, GSK3, PIM1, and DYRK1A) in the sub-nanomolar range. It is also toxic to cancer cells due to strong topoisomerase I inhibition. Lamellarins B and D differ only in the OH and OMe groups’ number and position on a common pyrrolo(2,1-*a*) isoquinoline scaffold (Figure 15). 

**Figure 16:**
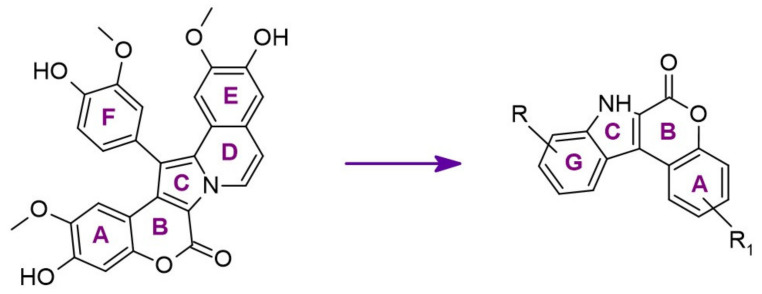
The main modification in lamellarin D rings.

The synthetic model for modulation of lamellarins’ activity has been developed [154]. Based on the natural structure’s fine-tuning, it was possible to eliminate topoisomerase affinity and cytotoxicity while retaining the kinase inhibition (Figure 16). The pyrrole moiety was replaced with an indole skeleton and designing new chromeno[3,4-b]indoles. The other parts of lamellarin D are unchanged (C, B, A,) as are the substituents most strongly interacting with the A ring, i.e., OH and -OCH_3_ groups. As a result of the presence of a hydroxyl group at position C-2, topoisomerase inhibition is lost. Interestingly, selective inhibition of DYRK1A was observed simultaneously. Without any other substituent or the addition of a hydroxyl group in C-10, two derivatives (4-hydroxychromeno(3,4-*b*)indol-6(7H)-one and 3-hydroxychromeno(3,4-*b*)indol-6(7H)-one) were selected with IC50 = 74 and 76 nM, respectively [154].

**Figure 17:**
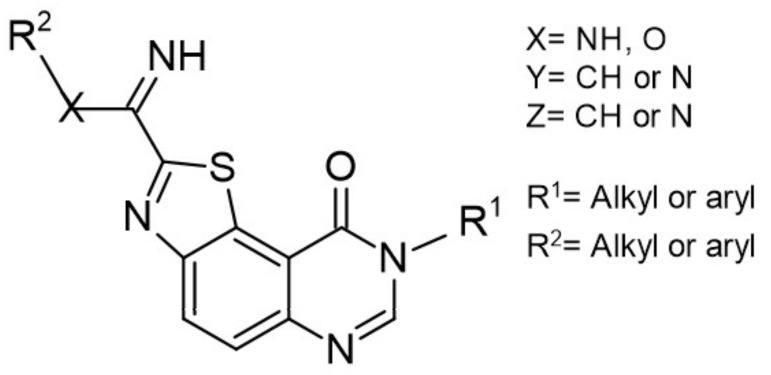
Chemical structures of 8H-thiazolo[5,4-*f*]quinazolin-9-ones derivatives.

Similarly, DYRK1A inhibitors comprising: (i) meridianines, (ii) indirubin 5′-carboxylates, (iii) thiazolo[5,4-f]quinazolines, (iv) pyrido[2,3-d]pyrimidines, (v) 3,5-diaryl-7-azaindoles (DANDYs), (vi) KH-CB19, (vii) 2,4-disubstituted thiophenes, and (viii) hydroxybenzothiophenes are tested not only towards DYRK1A selectivity but, additionally, towards structurally closely related kinase isoforms [155]. The Meijer group has been intensively investigating the C, N, S- or C, N, O-containing heterocycles representing precursors of biologically important molecules able to alter the kinases activity. One of the most promising compounds from this group is 8H-thiazolo[5,4-*f*]quinazolin-9-ones, with micromolar DYRK1A inhibitory potency (Figure 17). 

**Figure 18:**
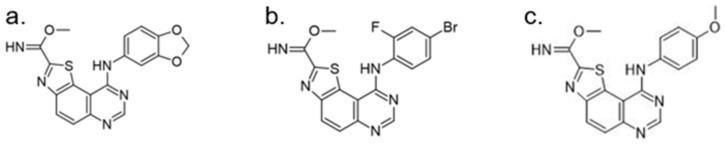
Chemical structures of (**a**) methyl 9-(4-methoxyphenylamino)thiazolo[5,4-*f*]quinazoline-2-carbimidate, (**b**) methyl 9-(benzo[d][1,3]dioxol-5-ylamino)thiazolo[5,4-*f*]quinazoline-2-carbimidate, and (**c**) methyl 9-(4-bromo-2-fluorophenylamino)thiazolo[5,4-*f*]quinazoline-2-carbimidate.

Benzo-, pyrido- and pyrazinothieno[3,2-d]pyrimidines derivatives as DYRK1A inhibitors were also investigated. Thiazolo[5,4-f]quinazoline scaffolds also indicate the potential for DYRK1A inhibition. Among the compounds of this library, methyl 9-(4-methoxyphenylamino)thiazolo[5,4-f]quinazoline-2-carbimidate, methyl 9-(benzo[d][1,3]dioxol-5-ylamino)thiazolo[5,4-f]quinazoline-2-carbimidate, and methyl 9-(4-bromo-2-fluorophenylamino)thiazolo[5,4-f]quinazoline-2-carbimidate inhibited DYRK1A with IC50 at nanomolar values (40, 47 and 50 nM, respectively) (Figure 18) [156,157]. 

**Figure 19:**
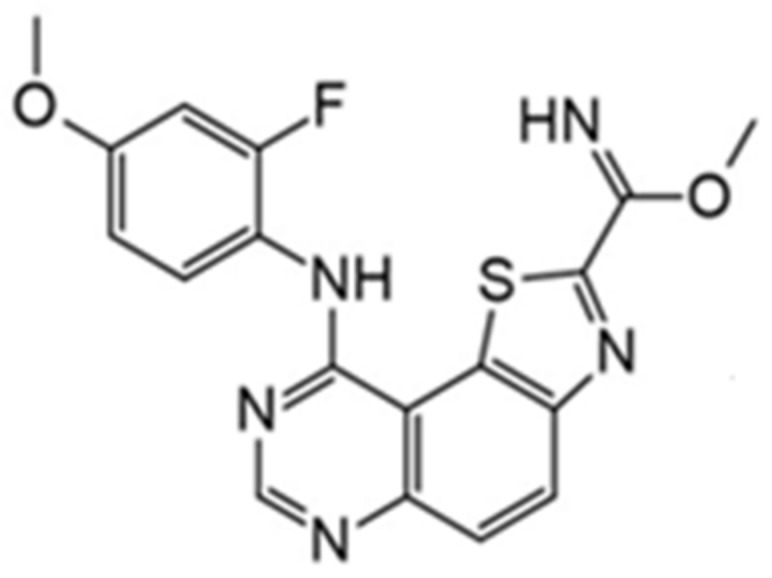
Chemical structure of EHT 5372.

It was also reported that the other derivative, the methyl 9-anilinothiazolo[5,4-*f*]quinazoline-2-carbimidate (EHT 5372), inhibits DYRK1A and DYRK1B at subnanomolar concentrations with IC50 = 0.22 nM for DYRK1A and 0.28 nM for DYRK1B, respectively). EHT 5372 and its derivatives are one most potent DYRK1A inhibitors reported so far, with high selectivity toward DYRK1A compared to other kinases of the CMGC group (Figure 19). EHT 5372 also inhibits cellular DYRK1A-mediated tau phosphorylation and Aβ production. However, it indicates significantly lower potency with IC50 1.06–1.17 µM [156,157].

**Figure 20:**
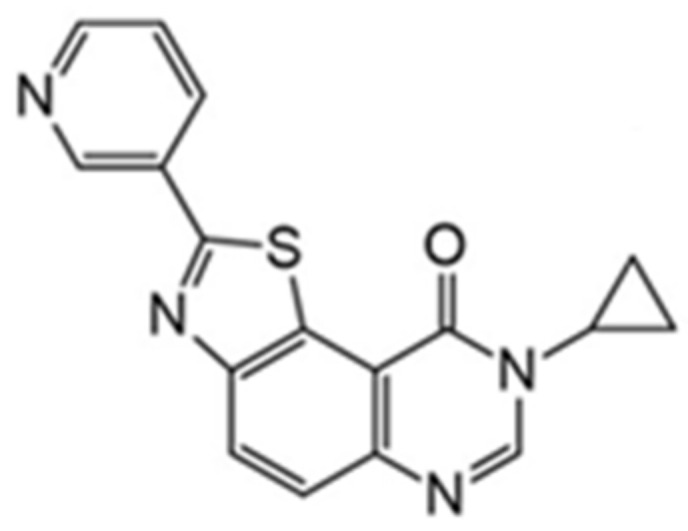
Chemical structure of FC162.

Another compound belonging to the DYRK1A inhibitor class and characterized by nanomolar IC50 values is 8-cyclopropyl-2(pyridin-3-yl)thiazolo[5,4-*f*]quinazolin-9(8H)-one (also called FC162, Figure 20) [158]. FC162 has emerged as the most promising candidate based on in vitro cell studies than well-characterized DYRK1A inhibitors (e.g., leucettine 41 and EHT1610). It was reported that FC162 could cross the BBB and is effective in Thr212 phosphorylation [158]. 

In further studies, the activity of FC162 on tau-4R cells (SH-SY5Y cells which overexpressing the four-repeat human tau isoform) was examined. The results indicated a dose-dependent inhibition of tau phosphorylation at Thr212. Moreover, the decrease in cyclin D3 phosphorylation at Thr283 was observed in murine pre-β-cells. After long-term treatment of FC162, a decreased G0 cell population was observed. Thus, these data reveal that FC162 phenocopies the effect of *Dyrk1a* genetic deletion [158]. Moreover, the following compound from this group—10-iodo-11H-indolo[3,2-*c*]quinoline-6-carboxylic acid (KuFal194)[155] also indicated an in vitro activity against DYRK1A with IC_50_ = 6 nM and considerable selectivity in comparison to DYRK1B and CLK1. Nevertheless, due to the low water solubility of KuFal194, further in vitro and in vivo studies should be performed with caution. It seems reasonable to use appropriate formulations that not only solve the problem of lipophilicity but, perhaps, by increasing stability, also improve other important parameters.

**Figure 21:**
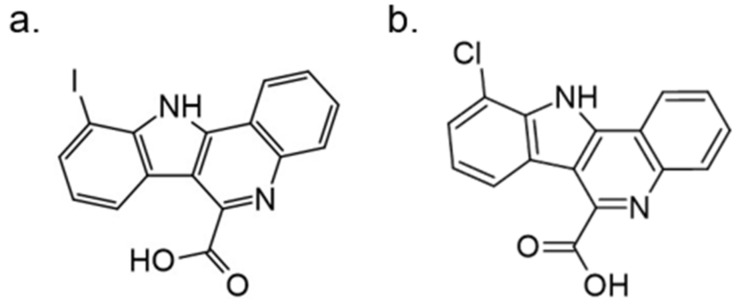
Chemical structures of (**a**) KuFal194 and (**b**) 10-chloro-11H-indolo[3,2-*c*]quinoline-6-carboxylic acid.

The SAR evaluation of KuFal194 derivatives and kinome selectivity analysis including DYRK1A and CMGC protein kinases: CDK1/cyclin B, CDK2/cyclin A, CDK5/p25, CK1, GSK-3, and ERK2 were performed. Substituents in the 8-position eliminated the DYRK1A inhibitory activity, suggesting steric exclusion from the ATP-binding pocket (Figure 21). Moderate, selective inhibitors of GSK3 were also obtained by adding polar H-bond acceptor substituents in 8-position. These showed no activity against DYRK1A. Strikingly, the 10-chloro derivative (10-chloro-11H-indolo[3,2-*c*]quinoline-6-carboxylic acid) showed two-fold higher DYRK1A inhibitory potency (IC50 = 31 nM) than 11H-indolo[3,2-*c*]quinoline-6-carboxylic acid without inhibiting other kinases [155].

**Figure 22:**
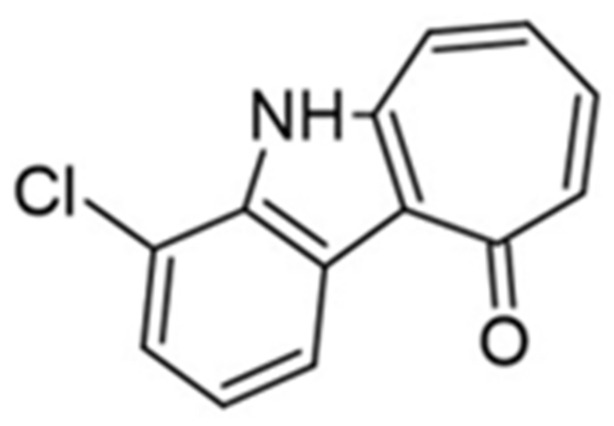
Chemical structure of 4-chlorocyclohepta[b]indol-10(5H)-one.

In order to enhance the physicochemical properties of KuFal194, a set of [b]-annulated chloro-substituted indoles were designed and developed. Compared to the iodine atom, the main rationale was that chlorine decreases the molar mass and lipophilicity and diminishes the overall toxicity [159]. The results of kinase inhibition studies performed using proper bioassays have revealed that most of the tested compounds, except Mannich base, act as DYRK1A inhibitors with micromolar or even sub-micromolar concentrations applied. Compared to KuFal194, these novel compounds were less active and non-selective towards CLK1 [158]. 4-chlorocyclohepta[b]indol-10(5H)-one was identified as a novel dual DYRK1A/CLK1 inhibitor with slightly better solubility. X-ray structure analysis confirmed the binding mode of this compound to DYRK1A, exploiting mainly shape complementarity for tight-binding (Figure 22) [158].

**Figure 23:**
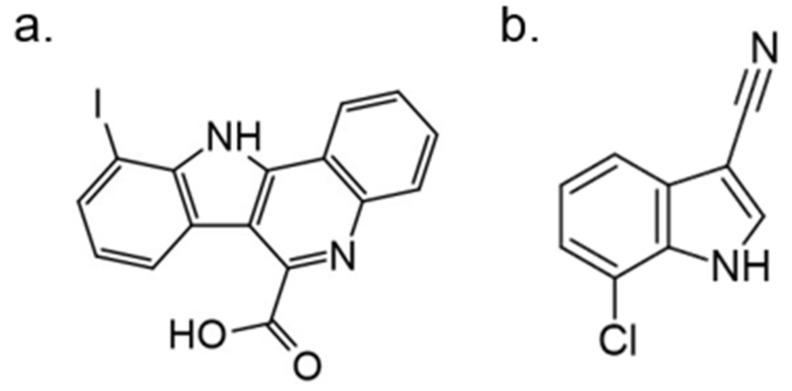
Chemical structures of (**a**) KuFal194 and (**b**) 7-chloro-1H-indole-3-carbonitrile.

In summary, inhibitors such as harmine, INDY, and leucine L41 have shown some promise in cellular assays due to their significant DYRK1A inhibitory activity. Results obtained against related kinases were no longer as promising, indicating their low selectivity. On the other hand, KuFal194 and EHT 5372 are characterized by proper selectivity against DYRK1A, but their use in in vivo studies is still limited due to high lipophilicity (Figure 23) [154]. Therefore, further design of improved water-soluble derivatives or the use of appropriate formulations is required.

**Figure 24:**
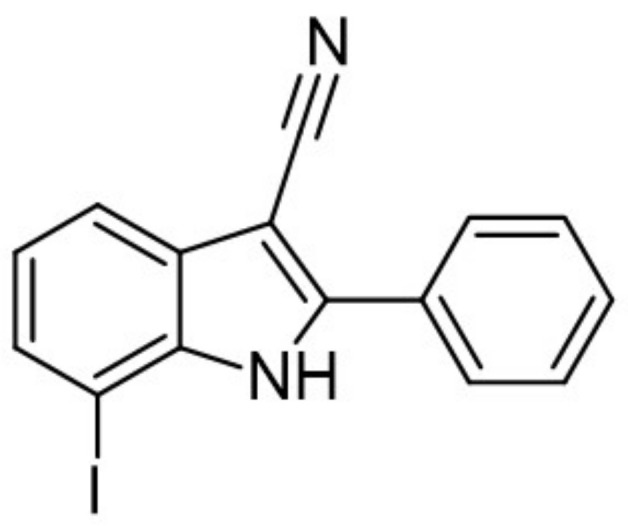
Chemical structure of 7-iodo-2-phenyl-1H-indole-3-carbonitrile.

A halogen-substituted indole group was chosen to develop more hydrophilic DYRK1A inhibitors with reduced molecular weight. The received fragment served as a template to design and develop a series of substituted indole-3-carbonitriles with inhibitory properties against CMGC kinases [160]. Computational studies indicated that the halogen substituents, at a 7 position of the indole ring, are most likely to interact with the hinge region by a water-mediated halogen bond [160]. At a 2 position of the indole core, only aromatic or lipophilic residues were tolerated. The 2-phenyl-substituted derivative (7-Iodo-2-phenyl-1H-indole-3-carbonitrile) was the most potent inhibitor of the series (IC50 against DYRK1A at 10 nM) and DYRK1A-mediated phosphorylation of SF3B1 in HeLa cells (IC50 = 320 nM) (Figure 24) [160]. However, it resulted in only low selectivity to related kinases of the CMGC group and poor aqueous solubility. To increase the solubility of the compounds, hydrophilic or aliphatic residues at a 2 position were introduced. By replacing the 2-phenyl substituent with pyridin-3-yl or cyclopentyl residues, the reduction of logP value and increased solubility were obtained, while the DYRK1A activity was only slightly affected. Further modifications of the 7-halogenindole-3-carbonitrile parent structure are underway to develop potent, highly selective, and water-soluble DYRK1A inhibitors [160].

**Figure 25:**
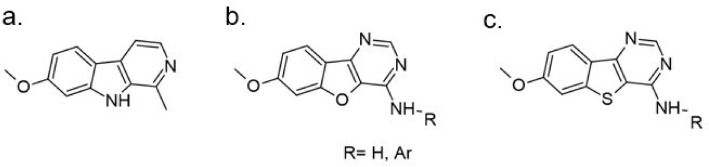
Chemical structures of (**a**) harmine, (**b**) N-aryl-7-methoxybenzo[b]furo[3,2-*d*]pyrimidin-4-amines, (**c**) N-arylbenzo[b]thieno[3,2-*d*]pyrimidine.

The tetracyclic V-shaped pyridine-, pyrazine- or indole-containing compounds represent the next set of molecules that target DYRK1A in the nanomolar range [12,161]. Inspired by the 6,5,6-fused tricyclic skeleton of harmine molecule, Meijer and Besson synthesized and characterized a series of N-aryl-7-methoxybenzo[b]furo[3,2-*d*]pyrimidin-4-amines and their N-arylbenzo[b]thieno [3,2-d]pyrimidine analogs substituted in position 4 of the pyrimidine ring by an aromatic amine (Figure 25). The kinase inhibition evaluation was carried out on Ser/Thr kinases, including CDK5, GSK3, DYRK1A, CLK1, and CK1. The benzothieno[3,2-d]pyrimidines derivatives-N-(2,3-dihydrobenzo[b][1,4]dioxin-6-yl)-7-methoxybenzothieno[3,2-*d*]pyrimidin-4-amine and N1-(7-methoxybenzothieno[3,2-*d*]pyrimidin-4-yl)-N4,N4-dimethylbenzene-1,3-diamine were found as a most active inhibitors submicromolar IC50 value and selectivity towards DYRK1A and CLK1 (0.5 and 0.68 nM, 0.7 and 0.66 for each inhibitor) [162]. 

**Figure 26:**
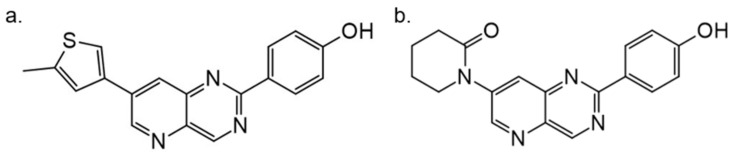
Chemical structures of (**a**) 4-[7-(5-methyl-thiophen-3-yl)-pyrido[3,2-*d*]pyrimidin-2-yl]-phenol and (**b**) 1-[2-(4-hydroxyphenyl)pyrido[3,2-*d*]pyrimidin-7-yl]piperidin-2-one.

Another study by Dehbi et al. reported the synthesis and biological evaluation of 4,7-disubstituted pyrido[3,2-d]pyrimidines. Using the SAR approach, the authors indicated that some of these compounds could selectively inhibit DYRK1A and CDK5 without affecting GSK3. The most active compound was 4-[7-(5-methyl-thiophene-3-yl)-pyrido[3,2-d]pyrimidin-2-yl]-phenol, which exhibited IC50: 110 nM for CDK5, 24 nM for DYRK1A and only 1.2 μM for GSK3. In the C-7 amino derivatives, the best was indubitably compound 1-[2-(4-hydroxyphenyl)pyrido[3,2-*d*]pyrimidin-7-yl]piperidin-2-one with IC50 = 60 nM against DYRK1A (Figure 26) [163]. These molecules have been tailored as dual selective DYRK1A and CDK5 kinase inhibitors involved in regulation processes, including CNS-related disorders like diabetic neuropathy [164].

**Figure 27:**
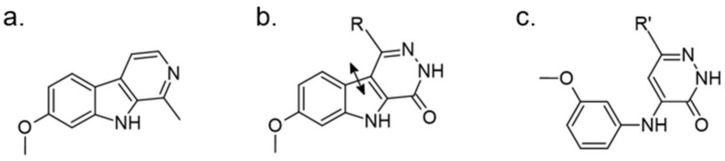
Chemical structures of pyridazino[4,5-b]indol-4-one scaffolds: (**a**) harmine; (**b**) and (**c**) general structures of synthesized compounds by Bruel et al.

Bruel et al. attempt to design new kinase inhibitors focused on a pyridazino[4,5-b]indol-4-one scaffold and found an inhibitor with micromolar IC50 (5 μM) against DYRK1A (Figure 27) [165]. Due to its structural analogy with harmine, further optimization was performed. The pyridazino[4,5-b]indol-4-one series, the furan-2-yl-substituted derivative, was selected as a compound with submicromolar IC50 (0.22 μM) against DYRK1A. It was only four-fold less active than harmine (0.06 μM) and indicated no activity towards the other kinases. The mechanism of its activity was explained theoretically. Based on the presented docking model, the authors suggested differences in its affinity towards harmine. Harmine interacts with the Leu241 residue via hydrogen bonding and the presented inhibitor probably bind to the pyridazinone ring (backbone atoms of Glu239 and Leu241). Furthermore, selectivity to CDK5 and GSK3 kinases may be due to an additional hydrogen bonding interaction between a methoxyl group and an aspartate residue (Asn244) located in the kinase pocket (Asp86 in CDK5; Thr138 in GSK3) [165].

**Figure 28:**
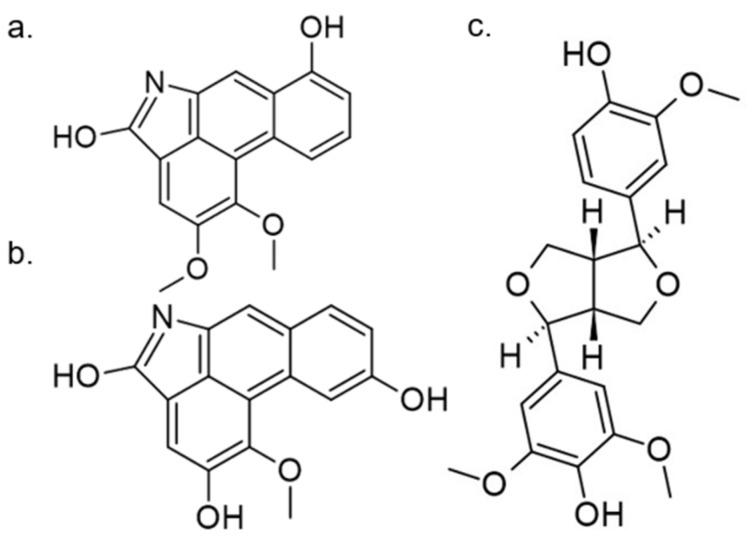
Chemical structures of (**a**) velutinam, (**b**) aristolactam AIIIA, and (**c**) medioresinol.

A systematic in vitro evaluation of 2500 plant extracts from New Caledonia and fromFrench Guyana was performed in another work. Aristolactams and lignan derivatives were purified from *Oxandra asbeckii* and *Goniothalamus dumontetii*. Porphine alkaloids were isolated from *Siparuna pachyantha*, *S. decipiens*, *S. guianensis,* and *S. poeppigii*. Among these compounds, velutinam, aristolactam AIIIA, and medioresinol showed submicromolar IC50 values on DYRK1A (Figure 28) [166].

#### 5.1.3. Azaindoles 

**Figure 29:**
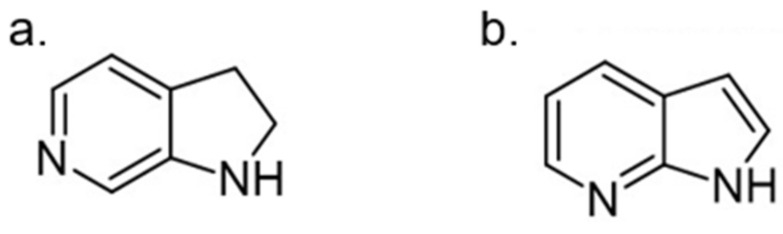
Chemical structures of (**a**) 6-azaindole and (**b**) 7-azaindole.

Azaindoles are structurally related to indoles, widely present in natural products and pharmaceuticals (Figure 29). Azaindole molecules appear to inhibit kinases than other targets preferentially. Moreover, their biological/pharmacological features are beneficial to treat many diseases, including DM [167].

**Figure 30:**
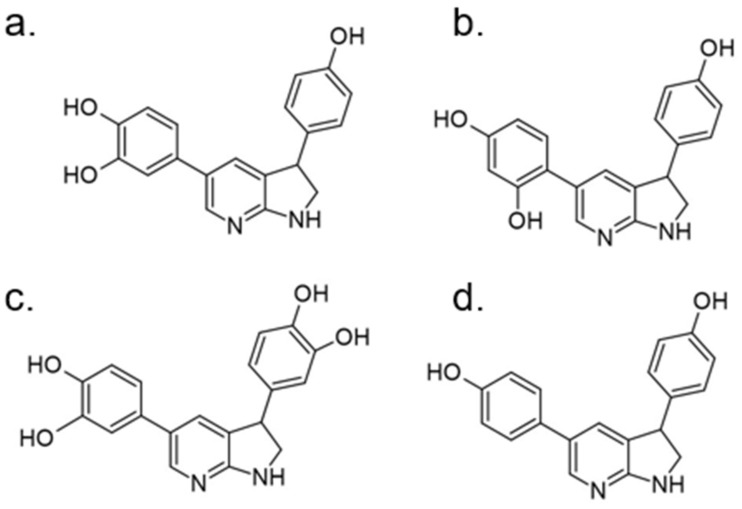
Chemical structures of DANDYs: (**a**) 3,5-di-(4-hydroxyphenyl)-1H-pyrrolo[2,3-*b*]pyridine; (**b**) 3-(4-hydroxyphenyl)-5-(2,4-dihydroxyphenyl)-1H-pyrrolo[2,3-*b*]pyridine; (**c**) 3,5-di-(3,4-dihydroxyphenyl)-1H-pyrrolo[2,3-*b*]pyridine; (**d**) 3-(4-hydroxyphenyl)-5-(3,4-dihydroxyphenyl)-1H-pyrrolo[2,3-*b*]pyridine.

With some azaindoles being successfully developed as antidiabetic drugs, the 6-azaindole and 7-azaindole derivatives have also been tested as DYRK1A inhibitors. For instance, the 3,5-diaryl-7-azaindole derivative, also called DANDY, represents one of the most potent inhibitors (IC50 = 3 nM) of DYRK1A. Besides DANDY, numerous DYRK1A inhibitor scaffolds have been reported (Figure 30) [168]. 

Based on molecular docking study at the ATP-binding site, it was demonstrated that there were multiple H-bond interactions with the peptide backbone (Glu239, Leu241) for the 7-azaindolecore, and the hydroxyl substituents interacted with Lys188 and Ileu165. The hydroxyl derivatives showed more remarkable activity than their methoxy derivatives [168]. Moreover, it was indicated that 6-azaindole derivatives were considerably less active than the 7-azaindole ones. Interestingly, when these derivatives were tested against a representative kinase panel, a relative selectivity appeared with compounds acting mainly on the DYRK1A family [169]. 

**Figure 31:**
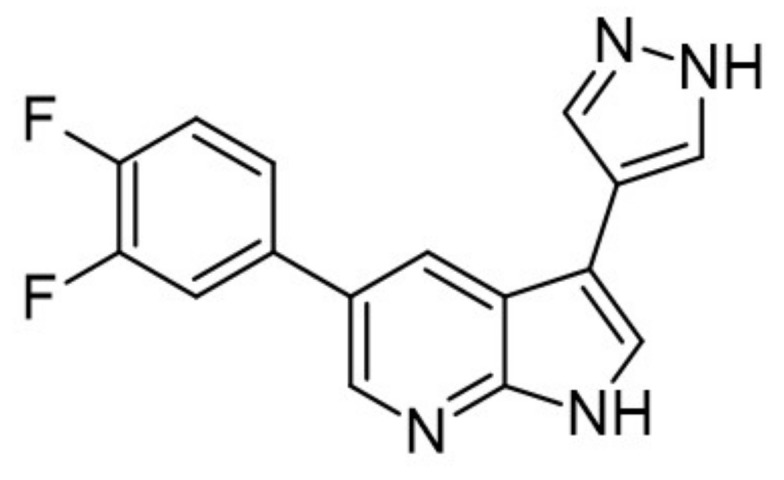
Chemical structure of GNF3809.

The SAR study of the azaindoles shows that the nitrogen at the 7-position is indispensable, as replacing the azaindole ring with the indole ring led to an inactive compound [170]. Methylation of the nitrogen at the N1-position of 7-azaindole indicates a similar effect. Thus, it suggests that the azaindole’s NH belongs to the pharmacophore. Furthermore, the addition of nitrogen to the azaindole ring led to a less active molecule. Moreover, additional substitution at the 2 position results in a significant decrease in its activity [170]. It appeared that the 7-azaindole core was indeed critical for the strong protective effect effect of INS-1E cells in the CK assay. Thus, 5-(3,4-difluorophenyl)-3-(pyrazole-4-yl)-7-azaindole (GNF3809) was selected for both, ex vivo and in vivo proof-of-concept efficacy studies (Figure 31) and demonstrated protective effects of β-cells. Future efforts directed at further optimization of GNF3809 and the elucidation of its molecular mechanism of action hold the substantial potential to address the unmet medical needs of T1D patients [170].

#### 5.1.4. Aminopyrazines

**Figure 32:**
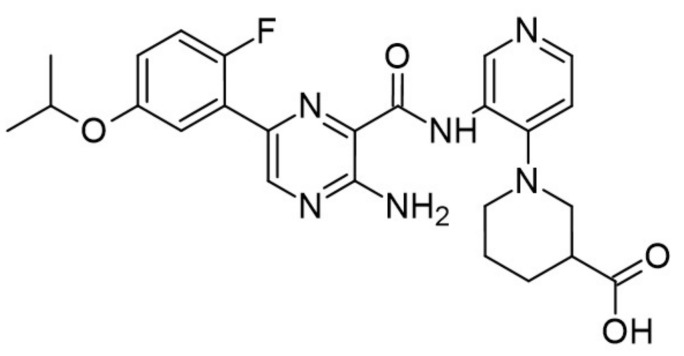
Chemical structure of GNF4877.

The aminopyrazine scaffold was identified from a phenotypic high-throughput screening campaign measuring β-cell proliferation using mouse R7T1 β-cells. Lead optimization results in identifying a promising dual DYRK1A and GSK3β inhibitor aminopyrazine GNF4877 (Figure 32) [171]. Priming of GSK3β substrates by DYRK1A has linked the former kinase and diabetes. The implication of these kinases in β-cell proliferation has been demonstrated via several screening tests and biological activity experiments. GSK3B action leads to NFAT nuclear localization. Inhibition of GSK3β is required for β-cell proliferation. The inhibition of DYRK1A may stimulate the NFAT signaling, which influences the β-cell proliferation.

SAR studies on the aminopyrazine scaffold targeted the enzymatic inhibition of DYRK1A using a structure-directed approach. GNF4877 is an inhibitor not only of DYRK1A but also of GSK3β. It affects the proliferation of β-cells in both in vitro and in vivo conditions. Nevertheless, inhibition of GSK3β may also lead to the appearance of some side effects. For this reason, GNF4877 was not selected for further clinical trials. Nevertheless, preclinical studies of this series of compounds have established a solid ground for the discovery of the next generation of selective DYRK1A inhibitors.

**Figure 33:**
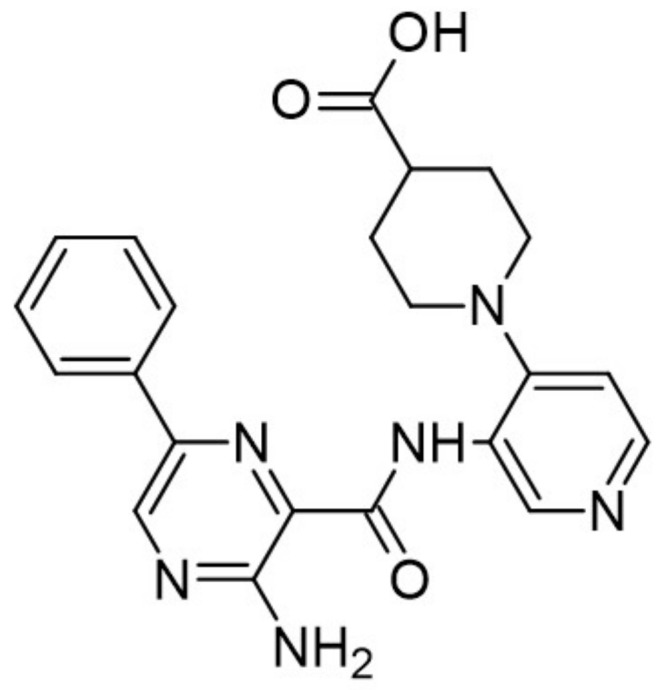
Chemical structure of GNF7156.

Aminopyrazine compounds (GNF series) were designed and developed to increase β-cell proliferation in adult primary islets. Oral administration of these compounds to diabetic mice induced β-cell proliferation and increased insulin content and consequently improved glycemic control. Biochemical, genetic, and in vitro studies confirmed that DYRK1 affects β-cell proliferation induced by GNF7156. Furthermore, dual-inhibition of DYRK1A and GSK3β increased β-cell proliferation (Figure 33) [171].

**Figure 34:**
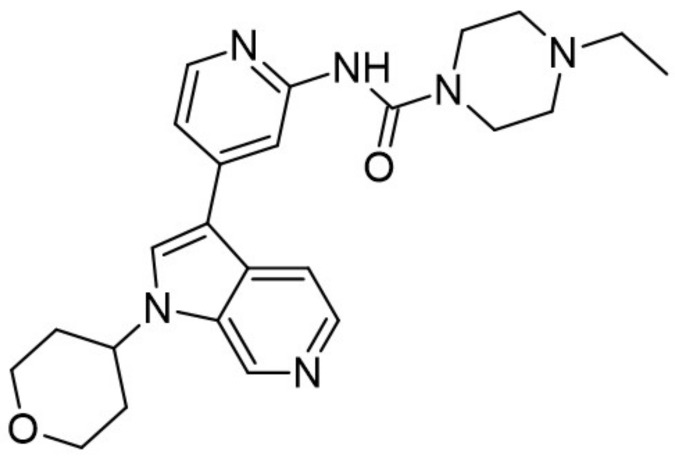
Chemical structure of GNF2133.

However, GSK3β regulates various cellular processes, including behavior, immunity, and circadian rhythm. Its inhibition may also activate other pathways and lead to undesired side effects. Less than a year ago, research into optimizing the structure and function of aminopyrazine led to the discovery and development of GNF4877 as a dual-function DYRK1A and GSK3β inhibitor of β-cell proliferation. Notably, compared to previously reported derivatives, this dual-mode agent was already active in nanomolar concentrations [171]. Another 6-azaindole derivative named GNF2133 has been developed as a DYRK1A inhibitor and has been shown to promote β-cell proliferation and restore its function (Figure 34). It was reported that the 6-azaindole was the most promising in DYRK1A inhibition and selectivity over GSK3β inhibition. It demonstrated significant dose-dependent glucose disposal function and insulin secretion in response to glucose potentiates arginine-induced insulin secretion (GPAIS) in rat insulin promoter and diphtheria toxin A (RIP-DTA) mice. Therefore, it should be concluded that it is an up-and-coming candidate for the treatment of type I diabetes [172].

**Figure 35:**
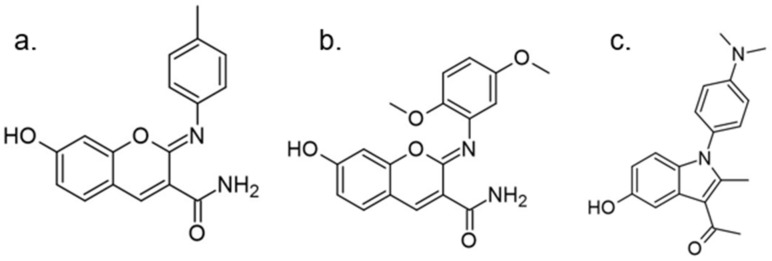
Chemical structures of (**a**) GNF-9228, (**b**) GNF-4088, and (**c**) GNF-1346.

Three novel compounds, GNF-9228, GNF-4088, and GNF-1346 (Figure 35), effectively stimulated β-cell proliferation, but not the expression of homeobox genes NKX6.1 or VGF, were described [173]. Subsequent studies demonstrated several salutary effects of the VGF prohormone and its encoded peptides, such as TLQP-21, on β-cell survival and function [173,174,175]. The most promising, GNF-9228, selectively activates human β-cell relative to α-cell proliferation and does not affect δ-cell replication [173]. GNF-9228 stimulates proliferation by a mechanism distinct from DYRK1A inhibitors because DYRK1A overexpression does not influence it and does not activate NFAT translocation [173]. In conclusion, a small molecule with pleiotropic positive effects on islet biology was characterized, including stimulation of human β-cell proliferation and insulin secretion and protection against multiple agents of cytotoxic stress [173].

#### 5.1.5. AC Inhibitors

**Figure 36:**
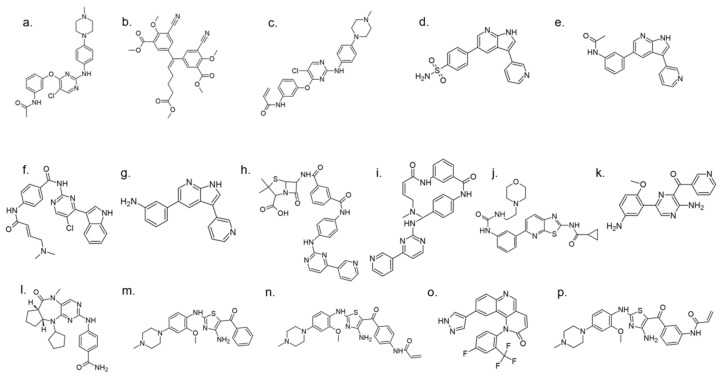
Chemical structures of AC compounds: (**a**) AC2 ***, (**b**) AC7 ***, (**c**) AC8 (**d**) AC12 *, (**e**) AC13 *, (**f**) AC14 *, (**g**) AC15 *, (**h**) AC16 ***, (**i**) AC18 ***, (**j**) AC20 ***, (**k**) AC22 ***, (**l**) AC23 ***, (**m**) AC24, (**n**) AC25, (**o**) AC27 *, (**p**) AC28. Range of the inhibition of the phosphorylation (peptide RRRFRPASPLRGPPK) for the compounds: * Ki =100–250 nM; *** 1.7 μM to >8 μM [177].

A set of DYRK1A inhibitors were identified by employing KINOMEscan [176] screening. These compounds, designated as AC, represent six different chemical scaffolds [177]. Compounds 12 and 15 share a 3-(3-pyridin-3-yl-1H-pyrrolo[2,3-b]pyridin-5yl)phenyl core with sulfonamide (para) or amine (meta) substituents on the terminal arene. Compounds 24, 25, and 28 are 4-[4-amino-2-[2-methoxy-4-(4-methylpiperazin1-yl)anilino]-1,3-thiazole-5-carbonyl]phenyl derivatives (Figure 36). Compound 24 constitutes the core scaffold, whereas 25 and 28 include terminal acrylamide functions added to the terminal arene at para and meta positions. Compound 28 lacks a methoxy substitution on the central phenyl ring. Compound 20 represents the other core scaffolds with 7-azathiazole, compound 22 as a pyrazine, 23 as an alkaloid, and 27 as a substituted 1,6-phenanthroline [177]. 

Selected compounds comprise a broad spectrum of biological activity towards DYRK1A kinase: from little to strong inhibition, measured by remaining activity (70−100% up to <5%). The measured Ki of the inhibition of the phosphorylation of DYRKtide (peptide RRRFRPASPLRGPPK) shows the variation among the compounds, with preservation of the chemical differences between the scaffolds [177].

Compounds 23 and 27 showed the highest activity in cellular assays at concentrations significantly lower than harmine. Comparing the activity of these two inhibitors with harmine, a 5-fold and 50-fold increase in activity was observed for 23 and 27 at concentrations of 1 µM and 0.1 µM, respectively. Excessive increase in the dose of the inhibitor in the cells leads to a decrease in activity, which may indicate a toxic effect, while dose-dependent inhibition is observed for harmine at these concentrations [177].

**Figure 37:**
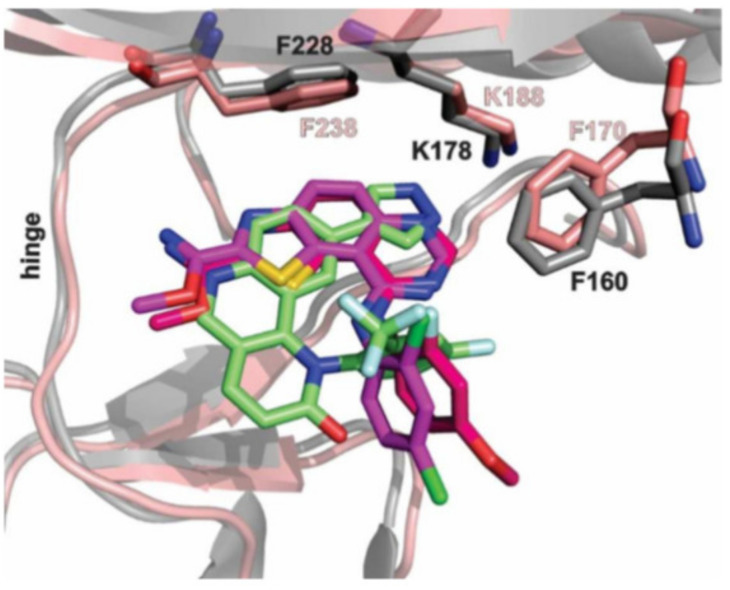
Comparison of the binding of AC27 (light green, PDB: 6EIS) with EHT1610 (red, PDB: 5LXD) and EHT5372 (magenta, PDB: 5LXC); (DYRK1A, salmon; DYRK2, gray) [177].

Moreover, this diverse set of scaffolds revealed the ability to prevent tau phosphorylation. Some of the inhibitors were co-crystallized with DYRK1A (12, 15, 24, 25, 28, 22, 27). The obtained crystal structures show that, with one exception, the inhibitors are typical hinge binders [177]. The most promising of the reported compounds from the AC series, 27, has no hydrogen bond to the hinge. It is a unique feature. Hydrogen bonds with K188 and E203 are formed to its diazole group and N244 via its carbonyl. Additionally, the N292 side chain forms a hydrogen bond with the fluorinated arene. Bridging water between the hinge and the compound was found in only one of the chains of the tetramer. It resides at a hydrogen-bonding distance of 2.7 Å from the 1,6-phenanthroline nitrogen, nearby (2.8 Å) the main chain nitrogen of L241 and 2.6 Å to the carbonyl of E239. The tri-fluoromethyl, fluorobenzyl ring is in perpendicular π-stacking with 1,6-phenanthroline and diazole rings. The trifluoromethyl group penetrates a hydrophobic pocket formed by G166 of the glycine-rich loop, I165 and V173 side chains. Compounds EHT1610 and EHT5372 (the most selective DYRK inhibitors identified so far) share remarkable similarities to compound 27. This suggests that the canonical hinge binding may be less critical for high affinity binding to DYRK2, as seen for this inhibitor. The benzyl rings of those scaffolds are roughly perpendicular to each other. While the overall orientation of the inhibitors differs, all three compounds interact with the P-loop. The trifluoromethyl moiety in 27 and the 2-fluoro- and 2-chlorobenzyl groups of EHT1610 and EHT5372 fill the same subpocket. The overall shape of these molecules can be described as “U” shaped. The opening of the “U” in AC27 is directed toward the P-loop (F160). This arrangement is reversed for the EHT inhibitors (Figure 37) [177].

Newly discovered binding features, such as CH-O interaction with Asn292 or binding water molecules that serve as catalytic lysine anchors, may provide valuable information for the optimization of these DYRK1A inhibitors and related kinases, which could be used in the future to treat not only diabetes but also neurodegenerative diseases, particularly Alzheimer’s Disease. The reported findings, once again, confirm the importance of a multidirectional approach in the search and development of new DYRK1A- inhibitors [177].

There is emerging evidence demonstrating a role for DYRK1A in diabetes and β-cell proliferation, which expands the potential for pharmaceutical applications of DYRK1A inhibitors. The diversity of the novel scaffolds and the binding modes determined by crystal structure and in vitro assays may lead to novel strategies for diabetes treatment. Small molecular inhibitors of DYRK1A developed in our group indicate specific and strong binding affinity with promising therapeutical applications. One of our inhibitors is a potential regulatory agent for restoring pancreatic β-cell mass, secretory and regulatory functions to the organ. Hence, one of the aims is to further optimize the development of such inhibitors, depicting the mechanisms involved in the progression of diabetes. We have shown that the AC inhibitors developed by us are able to potentiate the glucose-stimulated insulin secretion in cultured β-cells and isolated mouse islets of Langerhans. These results correlate with the inhibitory efficacy of the compounds against DYRK1A kinase selectivity and human β-cell proliferation. We assessed the AC27 inhibitor for its ex vivo activity in the freshly isolated pancreatic islets from mice. The results show that in both hiPSC-islets and isolated mouse islet models, AC27 significantly increased insulin secretion relative to untreated groups. Furthermore, this effect may be improved by co-addition of RepSox, a selective inhibitor of the TGF-β type 1 receptor, or LY364947, a selective ATP-competitive TGF-β receptor kinase I inhibitor. Among others, the pathogenesis of impaired GSIS observed in T2D can be alleviated by these molecules. Controlled, stable regulation of cell function at the molecular level is taking its toll in regenerative medicine. Stimulation of functional cell growth will be more promising assuming that small molecule-induced human β-cell proliferation is reachable in clinical practice. This set of studies provides proof-of-concept that small-molecule-induced human β-cell proliferation is achievable and lends considerable promise to the goals of regenerative medicine for diabetes treatment.

#### 5.1.6. Miscellaneous Scaffolds and Drug Combinations

In 2020, the novel DYRK1A inhibitor named KVN93 was identified. This tau kinase inhibitor interacts with DYRK1A by targeting the ATP-binding site in its active conformation when the activation loop is phosphorylated. It was investigated in Alzheimer’s Disease treatment as a compound able to regulate cognitive function, β-amyloid pathology, and neuroinflammation. The in vivo studies revealed that KVN93 improves long-term memory and reduces amyloid plaque levels in 5XFAD mice by increasing the Aβ degradation enzyme. KVN93 can modulate neuroinflammation in microglial cells by regulating TLR4/AKT/STAT3 signaling. The experiments carried out in wild-type mice injected with LPS confirmed that KVN93 treatment reduced microglial and astrocyte activation. These data suggest that KVN93 is a potential therapeutic DYRK1A inhibitor and is able to regulate (i) cognitive/synaptic function, (ii) Aβ plaque load, and (iii) neuroinflammatory reactions [178].

The studies described by Allegretti and co-authors revealed that the anticancer kinase inhibitor OTS167 may act as a structurally novel, remarkably potent DYRK1A inhibitor to induce human β-cell replication [179]. Despite the OTS167’s target promiscuity and cytotoxicity, the multidimensional compound optimization was performed to tailor kinase selectivity towards DYRK1A and reduce its cytotoxicity. Indeed, the series of 1,5-naphthyridine derivative characterization yielded several leads with exceptional DYRK1A inhibition and human β-cell replication promoting potencies but substantially reduced cytotoxicity. The results suggest that these compounds are the most potent human β-cell replication promoting molecules described and exemplify the potential purposefully leverage off-target activities of advanced stage compounds for the desired application [179].

**Figure 38:**
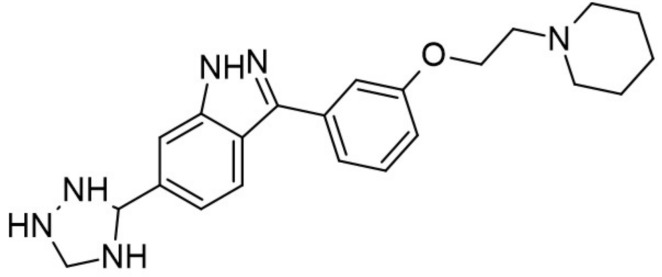
The chemical structure of CC-401.

In order to elucidate the molecular pathways that control β-cell growth, Abdolazimi et al. screened about 2400 bioactive compounds for rat β-cell replication-modulating activity [180]. In this library, the CC-401 was identified as a small molecule that promoted human β-cell replication (Figure 38). CC-401 is known as an advanced clinical candidate previously characterized as a c-Jun-N-terminal kinase inhibitor. However, these studies revealed that CC-401 also acts via DYRK1A/B inhibition [180]. Moreover, it was reported that DYRK1A/1B inhibition–dependent induction of β-cell replication is multifactorial. CC-401 treatment led to rodent (in vitro and in vivo) and human (in vitro) β-cell replication via DYRK 1A/1 B inhibition. In contrast to rat β-cells, which were broadly growth responsive to compound treatment (replication-inducing compounds like GSK3β or ALK5/TGFβ inhibitors), human β-cell replication was only consistently induced by DYRK1A/B inhibitors. In many reports, researchers identified the DYRK1A/B inhibition–dependent activation of NFAT as the primary mechanism of induction of β-cell–replication. Nevertheless, NFAT activity inhibition had a limited effect on CC-401–induced β-cell replication. Thus, the additional effects of CC-401–dependent DYRK1A/B inhibition were investigated. It has been found that CC-401 inhibited DYRK1A-dependent phosphorylation/stabilization of the β-cell–replication inhibitor p27Kip1. Additionally, CC-401 increased the expression of numerous replication-promoting genes generally suppressed by the dimerization partner, RB-like, E2F, and multi-vulval class B (DREAM) complex depends upon DYRK1A/B activity for integrity, including MYBL2 and FOXM1. These data demonstrate CC401 derivatives (abbreviated as STF compounds) and one of the commonly used DYRK1A inhibitors like harmine as a valuable resource for manipulating the signaling pathways that control β-cell replication and leverage DYRK1A/B inhibitors to expand understanding of the molecular pathways that control β-cell growth [180].

Additionally, the potential of combining small molecule inhibitors to augment the limited replication response of human β-cells was demonstrated. This effect was enhanced by simultaneous glycogen synthase kinase–3β (GSK3β) or activin A receptor type II-like kinase/transforming growth factor-β (ALK5/TGFβ) inhibition [30]. 

It was reported lately that the combination of inhibition DYRK1A with transforming growth factor-beta superfamily (TGFβSF)/SMAD signaling leads to a synergistic increase in human β-cell proliferation and the number of β-cells in both mouse and human islets. This effect is related to the activation of cyclins and CDKs with the decreased levels in key cell-cycle inhibitors (including CDKN1C and CDKN1A) through altering their Trithorax- and SMAD-mediated transactivation. Additionally, this dual DYRK1A and TGFβ inhibition allow the preservation of β-cell functions. These effects were proved in healthy human- and stem cell-derived β-cells as well as patients with T2D both in in vitro and in vivo investigations [181]. Furthermore, the relationship between DYRK1A and insulin receptor substrate-2 (IRS2) has been thoroughly discussed. The loss of IRS2 expression in β-cells contributes to T2D. It was also indicated that IRS2 might be one of the DYRK1A targets. DYRK1A directly interacts with IRS2 through the N-terminal domain of DYRK1A. Moreover, DYRK1A promotes tyrosine(Y)-phosphorylation and K48-linked poly-ubiquitination of IRS2 with the proteasomal degradation of IRS2. In vitro evaluation revealed the expression of DYRK1A in MIN6 cells and β-cells islets and pointed its role in inducing apoptosis. Furthermore, IRS2 expression was slightly reduced in the hippocampus and islets of young APP/PS1 mice (3-month-old), while it was significantly suppressed in older animals (6-month-old mice). It was postulated that it might be related to other mechanisms, e.g., activation of GSK3β and neuroinflammation in the early stage of the disease [182]. These findings also complement the current understanding of the relationship between DM and AD [182]. Some evidence was also provided that the combination of any GLP1R agonist class member with any DYRK1A inhibitor class member induces a synergistic increase in human β-cell replication accompanied by an increase in human β-cells mass [50]. A combination of small-molecule DYRK1A inhibitor (such as harmine, INDY, leucettine, 5-IT, GNF4877) to any one of the antidiabetic drugs that directly (GLP-1 analogs) or indirectly (DPP4 inhibitors) activate the GLP1R and convert the mitogenically inactive GLP1R agonists into potent β-cell proliferative agents [50]. Combining these two agents boosted human pancreatic β-cell proliferation and expanded β-cell mass in human cadaveric islets ex vivo. For instance, cadaveric human islets were transplanted into immunodeficient mice with diabetes induced by streptozocin, and the mice were then treated with the drug combination. The animals showed increased insulin production and improved glycemic control than mice treated with either compound alone or no treatment. Both phosphorylated NFAT and cAMP-PKA mediated the synergistic effect of the two molecules on pancreatic β cell expansion–dependent activation of cell cycle genes such as cyclin-dependent kinases and β-cell-specific genes (e.g., GLUT2, PDX1, and NKX6.1) [50,183]. The resulting proliferation rates exceeded those of DYRK1A inhibitors alone and may be in a range that could restore β-cell mass in people with T2D and T1D [50,149,183].

It is well known that the nature of diabetes is unlikely to be fully addressed by the modulation of any single target. The “paradigm shift” determines the research when complexity prevails, and radical specificity is no more the ultimate target. Simultaneous targeting of both DYRK1A kinase enhances the restoration of the β-cell population. Alternatively, the combinatory treatment with inhibitors, hypoglycaemic agents (glucagon-like peptide-1 (GLP-1) receptor agonists), and cell markers (e.g., TGF-β) improves the proliferation rate in human cadaveric β-cells. However, current inhibitors lack target specificity, with risks of adverse effects. Thus, the need to identify drugs that provide an accelerated human β-cell proliferation of improved specificity remains the priority. The development of inhibitors is frequently compromised by suboptimal pharmacokinetics. Evidence has recently emerged that simultaneous targeting of both DYRK1A and GSK3β may further benefit in restoring the insulin-producing β-cell population. Moreover, the recent studies on the DYRK family show the compensatory mechanism for DYK1A and DYRK1B synergistic effect on the proliferation of the β-cells in mammalian cell culture models.

## 6. Biological Effects of DYRK1A Inhibitors

### 6.1. Diabetes

As mentioned above, several DYRK1A inhibitors are able to enhance β-cell proliferation and improve insulin secretion and glucose homeostasis [16]. The gold standard in this research, harmine, may increase human β-cell proliferation in culture by ca. 2%. Nevertheless, DYRK1A inhibitors, including leucettine-L41 and INDY, indicate comparable proliferative potential, while 5-IT and GNF4877 were found to be 10-fold more potent.

Therefore, inhibition of DYRK1A is an important mechanism underlying β-cell proliferation and emphasizes that the diabetic kinome is a key target that can increase the mitogenic activity of β-cells. Following DYRK1A inhibitor treatment, proliferation is enhanced by induction and nuclear translocation of NFAT transcription factors that affect the cell cycle. Furthermore, it is suggested that DYRK1A inhibitors attract other targets involved in the stimulation of β-cell proliferation. Thus, the role of the diabetic kinome seems to be crucial for the future development of anti-diabetic strategies. Several studies reveal that each of these DYRK1A inhibitors also inhibits other kinases, particularly members of the CMGC family, including (i) cyclin-dependent kinase (CDK), (ii) mitogen-activated protein kinase (MAPK), (iii) glycogen synthase kinase-3 (GSK3), and (iv) CDC-like kinases notably: DYRKs, CLKs, GSKs [149]. Noteworthily, it can be speculated that each of them may be involved in human β-cell proliferation. Importantly, GSK3 (involved in insulin signaling and the replication of β-cells) may be recognized as the most prominent target of DYRK1A inhibitors because DYRK1A functions as a priming kinase for GSK3 signaling and plays a substrate role in preparation for GSK3 phosphorylation. The interaction of DYRK1A inhibitors with GSK3β has been shown to lead to β-cell proliferation in rodents [3]. Furthermore, it has been suggested that dual-mode inhibition of DYRK1A and GSK3β may contribute to the efficacy of the aminoprazine derivative GNF4877 [171].

GSK inhibitors (LiCl, 1-Akp) have also been shown to increase human β-cell proliferation from 0.17% to 0.71% [5]. However, DYRK1A inhibitors act in a dose-dependent manner, with proliferation peaking after treatment with the optimal inhibitor concentration and decreasing at higher doses. These are results suggesting interactions with other kinases/targets at higher doses [2,6,7]. Furthermore, off-target effects are not necessarily limited to protein kinases. 5-IT has also been found to be an adenosine kinase inhibitor, and its β-cell mitogenic capacity may be attributed to adenosine kinase inhibition [8]. It is also possible that DYRK1A inhibitors may affect targets other than kinases. Harmine not only inhibits DYRK1A in human β-cells but also reduces the abundance of SMAD proteins [6]. In addition, it acts as an MAO inhibitor [16,149].

Nevertheless, it can be stated that the mitogenic effects and enhanced proliferation mediated by DYRK1A inhibitors act as translocation of NFAT transcription factors to the nucleus, with the consequent transactivation of cyclins (cyclin A, CDKs) and repression of CDK-inhibitors such as p15INK4, p21CIP1, and p57KIP2 [149]. Other possible mechanisms involving DYRK1A, necessary for β-cell restoration are (i) phosphorylation and stabilization of p27KIP1, (ii) phosphorylation of D-cyclins and acceleration of their degradation; (iii) phosphorylation of the DREAM complex member, LIN52, enforcing cell cycle arrest; and (iv) phosphorylation of tau protein crucial for AD [149].

All these data indicate that regulation of DYRK1A kinase activity is an important mechanism underlying human β-cell proliferation. Other potential kinases and therapeutic targets capable of enhancing β-cell mitogenic activity are also indicated. Therefore, a better understanding of the diabetic kinome is crucial for the design and development of new and innovative, more potent and selective small molecules.

### 6.2. Other Diseases 

This review focuses on DYRK1A inhibitors developed for β-cell restoration and treatment of diabetes. However, it is worth noting that the development of DYRK1A inhibitors may be beneficial in the treatment of other diseases, including neurological disorders such as Alzheimer’s Disease(AD), Parkinson’s and Huntington’s diseases, Down Syndrome (DS) [79] and cancer [20].

#### 6.2.1. Neurological Disorders

DYRK1A has been implicated in neuronal development and many others related signaling pathways. In DS, the triplication of chromosome 21 results in ca.1.5-fold higher DYRK1A levels than the general euploid population. This DYRK1A overexpression has been linked to the cognitive deficits associated with Down Syndrome [184]. Moreover, through hyperphosphorylation of tau protein (Alzheimer’s Disease protein) and the formation of insoluble tau aggregates, DYRK1A is also involved in neurodegeneration and neuronal loss appearing in AD [185,186].

Therefore, a therapeutic strategy for cognitive deficits associated with DS, and ultimately AD, would involve controlled inhibition of brain DYRK1A activity [187]. Over the past few years, several DYRK1A inhibitors have been developed, most of which bind to the enzyme’s active ATP site. However, there are selected exceptions, such as epigallocatechin gallate (EGCG), an allosteric inhibitor of DYRK1A that improves cognition in Ts65Dn mice (a well-established in vivo model for DS [185,188]. It was also reported that T65Dn mice with a normalized DYK1A gene copy number (two copies) were characterized by a decrease in (i) senescent cells population in the hippocampus and cortex, (ii) cholinergic neurodegeneration, as well as (iii) APP that promotes the production of pathogenic Aβ, and tau levels, in comparison to Down Syndrome mice with three copies of DYRK1A [189]. These data indicated that DYRK1A inhibition and normalization of its level could reduce or delay AD neuropathology [189].

#### 6.2.2. Cancer

Both overexpression and downregulation of DYRK1A are associated with neurological defects, reflecting the extreme gene-dosage sensitivity of this protein. It was reported that DYRK1A could act as both an oncogene and a tumor suppressor [190]. DYRK1A works as a negative regulator of the cell cycle, and its dosage can direct cells toward proliferation or exit from the cell cycle. It may also promote the survival of malignant cells by inhibiting pro-apoptotic pathways since the loss of DYRK1A can activate p53 (the increased degradation of DYRK1A caused by p53 activation is mediated by MDM2, which was found to interact with and ubiquitinate DYRK1A, ultimately leading to its proteasomal degradation) [191,192]. DYRK1A likely plays a tumor type-specific role, so whether DYRK1A inhibition would promote or inhibit tumor cell growth depends on the tissue type and tumor microenvironment.Although DYRK1A is most widely characterized for its role in brain development, DYRK1A is overexpressed in various diseases, including many types of cancers, such as leukemia [193,194], pancreatic adenocarcinoma [195,196,197], and gliomas [198,199]. 

It was also reported that DYRK1A could positively regulate the STAT3/EGFR/Met signaling pathway in human EGFR wild-type NSCLC cells. In addition, DYRK1A inhibition (by siRNA or an inhibitor) increased the anticancer activity of AZD9291 (EGFR inhibitor, Osimertinib) NSCLC cells [200]. Furthermore, it was reported that inhibition of DYRK1A destabilizes EGFR and reduces EGFR-dependent glioblastoma growth [201]. It was indicated that DYRK1A reduces the level of Cyclin D1 by phosphorylating on Thr286, inducing the proteasomal degradation of Cyclin D1 and cell cycle G1 phase arrest. Furthermore, DYRK1A suppression can promote the degradation of EGFR and reduce the self-renewal capacity of glioblastoma cells [200,201]. Pozo et al. investigated the ability of harmine and INDY to inhibit GBM tumor growth and survival [201]. They suggested that DYRK1A functions upstream of SPRY2 to modulate EGFR lysosomal targeting. Phosphorylation of SPRY2 by DYRK1A decreases its inhibitory influence on FGF-induced MAPK activation. In glioblastomas, several members of the SPRY family are included in a transcriptome module associated with the EGFR amplification status in GBMs, suggesting that they could act as oncogenes. Thus, destabilization of EGFR by DYRK1A inhibition may be a potential therapeutic target for a subset of EGFR-dependent GBMs [201]. Another example is CX-4945 (silmitasertib), a casein kinase 2 inhibitor currently in clinical testing for various cancers [202]. It was subsequently found to also potently inhibit several members of the CLK and DYKRK families, including DYRK1A, and was able to block DYRK1A-related tau phosphorylation in a mouse model of Down Syndrome.

## 7. Summary

Diseases related to diabetes and obesity are one of the major threats to human life. According to WHO, approximately 300 million people will be obese in 2035 [203]. This ever-increasing trend is difficult to prevent due to changing lifestyles around the world and energy-rich diet availability. Only in 2015, more than 1.6 million human deaths were caused by hyperglycemia and diabetes. Type 2 diabetes is now treated with various pharmaceuticals, but in fact there is no effective treatment. Other types of diabetes rely solely on supplementing the body with external insulin. Despite significant advances in insulin-based and other therapies, patients with diabetes will continue to receive medication throughout their entire lives. This causes an enormous healthcare burden and limits the comfort of patient’s life. 

Two main types of diabetes—T1D and T2D—share similar mechanisms of β-cell-function failure via an insufficient mass of the endocrine pancreatic cell fraction. In T1D, this phenomenon is driven by autoimmune assault against own cells, while T2D is characterized by insulin resistance and subsequent β-cell mass decrease. In general, T1D and T2D are, by definition, a blood hyperglycemia condition caused by total or relative deficits in β-cell mass. Existing therapies improve glycemic control but provide only a temporal relief, with lifetime dependency. Several early prevention measures and strategies offered for diabetic patients of T2D put this disease into reasonable control to delay the clinical onset. Such interventions do not exist for T1D. As stated by The Global Report of the World Health Organization (WHO), T1D cannot be prevented with current knowledge. Although effective approaches are available to prevent T2D, no cure for advanced disease is available. The optimal approach should reverse its pathologic changes to provide a cure rather than a lifetime pharmaceutical supplementation. Thus, finding an accurate cure for diabetes is of critical importance. Restoring metabolic homeostasis would free the patient from constant reliance on pharmaceuticals and monitoring glucose level. Nevertheless, so far, all the possible therapies are only in very early preclinical stages. The treatment strategies rely mainly on promoting β-cells differentiation. This promising strategy requires selective alteration of cellular differentiation to obtain a new, regenerated population of β-cells. Unfortunately, direct alteration of transcription factors is complicated, and there is no efficient strategy to affect the pancreas selectively. Therefore, more upstream biomolecular targets are sought. The importance of targeting protein kinases with small molecules is an irrefutable and great tool to establish therapeutical pathways to understand disease mechanisms. In particular, the finding of DYRK1A, a crucial protein kinase that has been implicated as a potential regulator of β-cells, raises its potential application in diabetes. DYRK1A is involved in cellular processes related to the proliferation and differentiation of β-cells. Thus, DYRK1A is one of the most extensively studied targets for β-cells regeneration. The β-cells differentiation observed when DYRK1A kinase activity is modulated points to a possibility of using “diabetic kinome” as a target for future DM therapies. Scientific investigations and the pharmaceutical industry have confirmed the role of DYRK1A kinase in various molecular processes. This review aims to highlight the knowledge and approaches taken under action within the past few years. These last five years have brought progress and even more questions about the actual position of the approach for many scientific fields. We present recent developments in diabetic kinome inhibitors, with a particular focus on DYRK1A. 

We have paid particular attention in this review to the fact that no DYRK1A inhibitors, to date, have met the selectivity standards needed for use as probe molecules. Harmine, one of the most commonly used inhibitors in DYRK1A-related research, possesses strong cross inhibition of monoamine oxidase (MAO), which would cause some adverse effects. The low selectivity also makes harmine unsuitable as a probe to test DYRK1A inhibition in cell lines. Efforts to eliminate the MAO inhibition while keeping DYRK1A inhibition led to the harmine derivative AnnH75. Another DYRK1A inhibitor, green tea flavonol epigallocatechin-gallate (EGCG), was shown to correct cognitive deficits in Down Syndrome mouse models and humans. However, it also potentially has multiple targets (and correspondingly is under consideration for use in a broad range of disorders) and cannot be considered a DYRK specific inhibitor. Thus, structural modifications may be introduced to achieve high selectivity. The so-called “gatekeeper” identity was identified early as a principal determinant of inhibitor selectivity. This residue initiates the “hinge” segment that links the two folding lobes of protein kinases, and its side chain lies adjacent to ATP inhibitors that bind via hydrogen bonding to the hinge. DYRK protein kinase targets consist of phenylalanine, which simultaneously offers good opportunities for inhibitor design and polypharmacology. Another opportunity for selectivity and favorable binding kinetics is covalent binding to sulfhydryl groups. The cysteine in the HCD (histidine cysteine aspartate) motif is the most prominent target for the DYRK1A. A third opportunity involves linking ATP-site inhibitors to peptides corresponding to substrate recognition sequences. This allows for high potency and selectivity for research compounds. In order to briefly summarize all the DYRK1A inhibitors discussed in this review, their IC50 values, targets, biological activity with future direction of development are listed in Table 2.

Compared to the well-known and the best to date inhibitor for increasing human pancreatic β-cell replication, the advantages of the newly identified fragments give us a privileged position in the race to the new therapeutics. Future studies should provide proof-of-concept that small-molecule–induced human β-cell proliferation is achievable with the use of regenerative medicine for diabetes therapy. The generation of the iPSC-derived β-cells has been one of the most desired strategies, with several protocols being invented. Functional iPSC-derived β-cells bring real hope for diabetic patients, who are not qualified for transplantation, with severe glycemic lability, recurrent hypoglycemia, and a reduced ability to sense symptoms of hypoglycemia (reduced hypoglycemia awareness). Providing an unlimited source of autologous, engineered cells from the somatic pool could significantly shift the availability of transplants from very limited to plentiful. Therefore, every finding and improvement in the prolonged intervention of diabetes is of the highest value. Current knowledge on the transplantation of the pancreatic islets tackles the severe problem of engraftment and stable implantation of the delivered cell mass into the organ. The importance of resolving this issue has been demonstrated broadly, and multiple methods are proposed to alleviate the problem. We would also like to propagate the term “diabetic kinome” within scientific terminology to emphasize the role of multiple kinases’ synergistic action in directing molecular processes that underlie this particular set of diseases. The human kinome constitutes over 500 kinases, responsible for every biological function and regulation in the cell. Therefore, finding the optimal selectivity profile for kinase inhibitors is of essential importance. 

## Figures and Tables

**Table 1:** Classification of diabetes mellitus by etiology [33].

Type	Etiology
type I diabetes	Immunologically-mediated Idiopathic
type II diabetes	Genetic predisposition, Insulin resistance (aging, physical inactivity, and overweight) Relative insulin deficiency and decreased β-cell function
gestational diabetes	β-cell dysfunction on a background of chronic insulin resistance during pregnancy
other types of diabetes	Genetic defects of β-cell function: • MODY 3, MODY 2, MODY 1 and others • transient and permanent neonatal diabetes • mitochondrial DNA and others Genetic defects in insulin action: • type A insulin resistance • leprechaunism, Rabson-Mendenhall syndrome • Lipoathropic diabetes and others The disease of the exocrine pancreas: • pancreatitis, trauma/pancreatectomy, neoplasia, cystic fibrosis, hemochromatosis, fibrocalculous pancreatopathy, and others Endocrinopathies: • acromegaly, Cushing’s syndrome, glucagonoma, pheochromocytoma, hyperthyroidism, somatostatinoma, aldosteronoma Drug or chemical-induced: • e.g., vacor, pentamidine, nicotinic acid, glucocorticoids, thyroid hormone, diazoxide, β-adrenergic agonists, thiazides, Dilantin, γ-IFN Infections: • congenital rubella • cytomegalovirus and others Uncommon forms of immune-mediated diabetes: • Stiff-man syndrome • anti-insulin receptor antibodies and others Other genetic syndromes sometimes associated with diabetes • Down syndrome, Klinefelter syndrome, Wolfram syndrome, Friedreich ataxia, Huntington chorea, Laurence-Mood-Biedl syndrome, myotonic dystrophy, porphyria, Prader-Willi syndrome and others

**Table 2:** IC50 value determined for DYRK1A inhibitors mentioned thorough this review. Abbrev. In order: ↑↑/↑↑↑ = moderate/most potent.

No.	Compound	Name	IC50 DYRK1A[nM]	Other Targets	In Vitro Results	Future Directions
1.	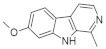	harmine	33	MAO-A CK1 PIM3	↑↑↑	increasing the selectivity of the compound
2.	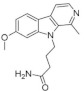	4-(7-methoxy-1-methyl-β-carbolin-9-yl)butanamide	25	MAO-A	↑↑↑	the research direction is definitely worth pursuing, large-scale clinical trials are needed
3.	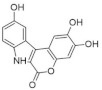	4-hydroxychromeno[3,4-*b*]indol-6(7H)-one	0.074	CDK5 GSK3	not available	results will be use to the chromeno[3,4-b]indole as a pharmacophore
4.	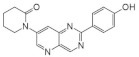	1-[2-(4-hydroxyphenyl)pyrido[3,2-*d*]pyrimidin-7-yl]piperidin-2-one	60	CDK5 GSK3	not available	promising scaffolds for targetingprotein kinases involved in the central nervous system
5.	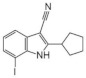	2-cyclopentyl-7-iodo-1H-indole-3-carbonitrile	70	CLK1 CLK2 CLK4 GSK3	minimal cytotoxicity, more data not available	further modifications are underway, aiming at the development of potent, highly selective and water-soluble DYRK1A inhibitors
6.	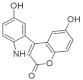	3-hydroxychromeno[3,4-*b*]indol-6(7H)-one)	500	CDK5 GSK3	not available	results will be used to the chromeno[3,4-*b*]indole as a pharmacophore
7.	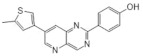	4-[7-(5-methyl-thiophen-3-yl)-pyrido[3,2-*d*]pyrimidin-2-yl]-phenol	24	CDK5 GSK3	not available	promising scaffolds for targetingprotein kinases involved in the central nervous system
8.	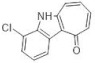	4-chlorocyclohepta[b]indol-10(5H)-one	200	CLK1	not available	biological data are needed
9.	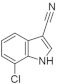	7-chloro-1H-indole-3-carbonitrile	3300	CLK1 CLK2 CLK3 CLK4 GSK3	not available	use for the development of new DYRK1A inhibitors
10.	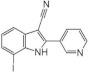	7-iodo-2-(pyridin-3-yl)-1H-indole-3-carbonitrile	80	CLK1 CLK2 CLK4	not available	biological data are needed
11.	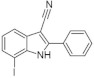	7-iodo-2-phenyl-1H-indole-3-carbonitrile	10	CLK1 CLK2 CLK3 CLK4 GSK3	minimal cytotoxicity, more data not available	biological data are needed
12.	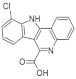	10-chloro-11H-indolo[3,2-*c*]quinoline-6-carboxylic acid	23	CLK1 CLK2 CLK3 CLK4	not available	biological data are needed
13.	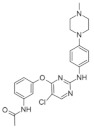	AC2	>16000	not available	not available	new scaffolds offer novel opportunities to design DYRK1A inhibitors
14.	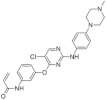	AC8	>8000	not available	not available	new scaffolds offer novel opportunities to design DYRK1A inhibitors
15.	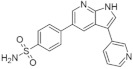	AC12	216	pan kinase	not available	new scaffolds offer novel opportunities to design DYRK1A inhibitors
16.	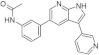	AC13	350	not available	not available	biological research in progress
17.	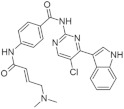	AC14	221	not available	not available	new scaffolds offer novel opportunities to design DYRK1A inhibitors
18.	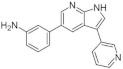	AC15	329	pan kinase	not available	new scaffolds offer novel opportunities to design DYRK1A inhibitors
19.	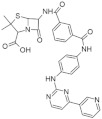	AC16	>6000	not available	not available	new scaffolds offer novel opportunities to design DYRK1A inhibitors
20.	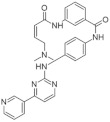	AC18	>4800	not available	not available	new scaffolds offer novel opportunities to design DYRK1A inhibitors
21.	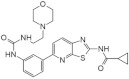	AC20	3500	CLK2 ABL PDGFR	not available	new scaffolds offer novel opportunities to design DYRK1A inhibitors
22.	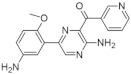	AC22	>6000	GSK3β CLK2 HIPK1/2 CDK7	not available	biological research in progress
23.	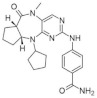	AC23	4200	DRAK1/2 ERK5 a	not available	biological research in progress
24.	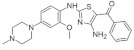	AC24	800	CLK2	not available	new scaffolds offer novel opportunities to design DYRK1A inhibitors
25.	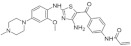	AC25	1200	CLK2	not available	biological research in progress
26.	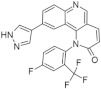	AC27	532	GSK3β PIK3CG PIK4CB	not available	biological research in progress
27.	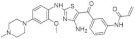	AC28	-	CLK2	not available	biological research in progress
28.	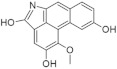	Aristolactam AIIIA	80000	CDK1 CDK2 CDK4	not available	possible use in viral infections, cancer and neurodegenerative pathologies (e.g., Alzheimer’s and Parkinson’s diseases)
29.	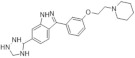	CC-401	-	c-Jun-N-terminal kinase PRKD2 PRKD3 CSNK1G3 MAPK9	↑↑	key issues are the development of strategies to target regenerative compounds selectively to the β cell
30.	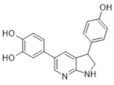	3,5-di-(4-hydroxyphenyl)-1H-pyrrolo[2,3-*b*]pyridine	3	Akt CDK2 CDK5 CK1 CLK1 ERK2 GSK3 JAK3 TRKA pim 1 kinase	Noncytotoxic, more data not available	studies on appropriate animal models
31.	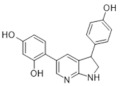	3-(4-hydroxyphenyl)-5-(2,4-dihydroxyphenyl)-1H-pyrrolo[2,3-*b*]- pyridine	11.7	Akt CDK2 CDK5 CK1 CLK1 ERK2 GSK3 JAK3 TRKA pim 1 kinase	Noncytotoxic, more data not available	studies on appropriate animal models
32.	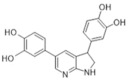	3,5-di-(3,4-dihydroxyphenyl)-1H-pyrrolo[2,3-*b*]pyridine	12.4	Akt CDK2 CDK5 CK1 CLK1 ERK2 GSK3 JAK3 TRKA pim 1 kinase	Noncytotoxic, more data not available	studies on appropriate animal models
33.	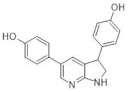	3-(4-hydroxyphenyl)-5-(3,4-dihydroxyphenyl)-1H-pyrrolo[2,3-*b*]- pyridine	23.1	Akt CDK2 CDK5 CK1 CLK1 ERK2 GSK3 JAK3 TRKA pim 1 kinase	Noncytotoxic, more data not available	studies on appropriate animal models
34.	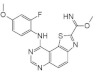	EHT5372	0.22	CLK1 CLK2 CLK4 GSK3	not available	high-potential therapy for AD and other tauopathies
35.	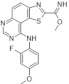	EHT1610	0.36	CLK1 CLK2 CLK4 GSK3	not available	high-potential therapy for AD and other tauopathies
36.	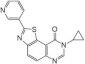	FC162	11	CLK1 GSK3	FC162 modified Tau phosphorylation and could alter cell cycle progression of pre-B cells	development as a DYRK1A kinase inhibitor
37.	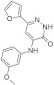	furan-2-yl-substituted	>10000	CDK5/p25 GSK3α/β PI3Kα	cytotoxic effects was determined against six human cancer cell lines	candidate for further evaluations
38.	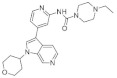	GNF2133	6	GSK3	↑↑	requires strategies to mitigate the observedhypertrophic effects in nonpancreatic tissues
39.	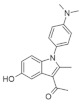	GNF1346	-	undetermined	↑	for now, untested
40.	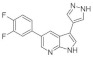	GNF3809	-	CDK CLK MAP4K4 GSK3 FLT HIPK JAK3	↑↑	further optimization and elucidation of its molecular mechanism of action needed
41.	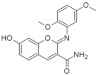	GNF4088	-	undetermined	↑	for now, untested
42.	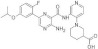	GNF4877	6	GSK3	↑↑	GNF4877 was notprogressed beyond preclinical research
43.	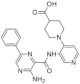	GNF7156	100	GSK3	↑↑	may provide a path forward to develop new drugs to treat diabetes
44.	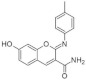	GNF9228	-	undetermined	↑↑↑	development of chemically modified versions of GNF9228 with enhanced bioavailability to *allow* in vivo testing
45.	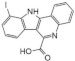	Kufal194	6	CLK-1	data available for zebrafish	promising starting point for the development of therapeutics in DS
46.	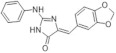	L41	170	CLKs mTOR/PI3K	not available	biological research in progress, promising compound forthe development of novel AD therapeutic agents
47.	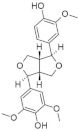	medioresinol	100	CDK1	not available	for now, untested
48.	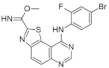	methyl 9-(4-bromo-2-fluorophenylamino)thiazolo[5,4-*f*]quinazoline-2-carbimidate	50	CK1 CDK5 GSK3	not available	a promising source for the synthesis of novel bioactive molecules
49.	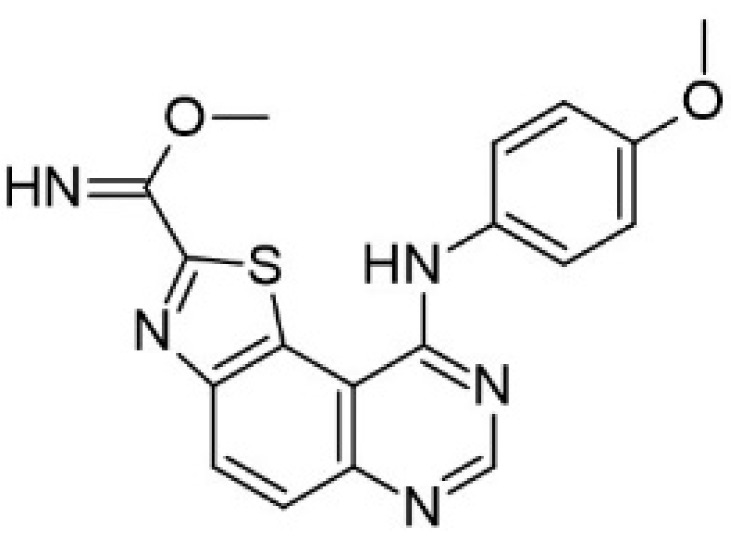	methyl 9-(4-methoxyphenylamino)thiazolo[5,4-*f*]quinazoline-2-carbimidate	40	CK1 CDK5 GSK3	not available	a promising source for the synthesis of novel bioactive molecules
50.	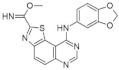	methyl 9-(benzo[d][1,3]dioxol-5-ylamino)thiazolo[5,4-*f*]quinazoline-2-carbimidate	47	CK1 CDK5 GSK3	not available	a promising source for the synthesis of novel bioactive molecules
51.	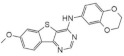	N-(2,3-dihydrobenzo[b][1,4]dioxin-6-yl)-7-methoxybenzothieno[3,2-*d*]pyrimidin-4-amine	0.5	TRPV1 CLK1	not available	starting point of a larger program
52.	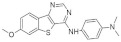	N1-(7-methoxybenzothieno[3,2-*d*]pyrimidin-4-yl)-N4,N4-dimethylbenzene-1,3-diamine	0.68	CLK1	not available	starting point of a larger program
53.	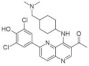	OTS167	-	undetermined	cytotoxic	a model compound for the design of less toxic compounds
54.	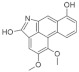	velutinam	600	CDK1	not available	for now, untested

## Abbreviations

AD	Alzheimer’s Disease
Akt	Protein kinase B
AMPK	5′AMP-activated protein kinase
BBB	Blood-brain barrier
β-FGF	Fibroblast growth factor
cAMP	3′,5′-cyclic adenosine monophosphate
CDKLs	CDK-like kinases
CDKN	Cyclin-dependent kinase inhibitor
CDKs	Cyclin-dependent kinases
CK2	Casein kinase 2
CLKs	CDC-like kinases
CMGC	Kinase family is named after the initials of its subfamily members, including cyclin-dependent kinase (CDK), mitogen-activated protein kinase (MAPK), glycogen synthase kinase (GSK) and CDC-like kinase (CLK)
c-Myc	Family of regulator genes and proto-oncogenes that code for transcription factors
CNS	Central nervous system
CREB	cAMP response element-binding protein
DM	Diabetes mellitus
DN	Diabetic Neuropathy
DPP-4	Dipeptidyl peptidase-4
DREAM	Dimerization partner, RB-like, E2F, and multi-vulval class B
DS	Down Syndrome
DSCR1 gene	Down Syndrome critical region gene 1
DYRK1A	Dual-specificity tyrosine phosphorylation-regulated kinase A
EGCG	Epigallocatechin gallate
ERK/MAPK	A chain of proteins in the cell that communicates a signal from a receptor on the cell’s surface to the DNA in the nucleus. The pathway includes many proteins, including MAPK (mitogen-activated protein kinases, originally-called ERK, extracellular signal-regulated kinases)
FDA	Food and Drug Administration
FKHR	Forkhead protein
FOX	Forkhead box
FOXM1	Forkhead Box M1
GAD65	Glutamic acid decarboxylase
GADA	Glutamic acid decarboxylase antibodies
GDM	Gestational diabetes mellitus
GLP-1	Glucagon-like peptide-1
GLUT4	Glucose transporter type 4
GPAIS	Glucose potentiates arginine-induced insulin secretion
GSK3	Glycogen synthase kinase 3
GSKs	Glycogen synthase kinases
HbA1c	Glycated hemoglobin
HK	Hexokinase
HLA	Human leukocyte antigen
HNF-1α	Hepatic transcription nuclear factor
HTS	High-throughput screening
IDE	Insulin-degrading Enzyme
IGF-1R	Insulin-like growth factor 1 receptor
IKKβ	I kappa beta kinase
IL-1β	Interleukin 1 beta
IL-12	Interleukin 12
IR	Insulin receptor
IRS1	Insulin receptor substrate 1
IRS2	Insulin receptor substrate-2
JNK	Jun N-terminal kinase
MAO	Monoamine oxidase
MAPK	Mitogen-activated protein kinase
MODY	Maturity-onset diabetes of the young
mTOR	Mammalian target of rapamycin kinase
MYBL2	Myb-related protein B
NFAT	Nuclear factor of activated T-cell
NKX6.1	Protein that in humans is encoded by the NKX6-1 gene
NME1/2	Nucleoside diphosphate kinase 1 and 2
p53	tumor protein P53
PI3K	Phosphatidylinositol 3-kinase
PI3K-AKT/PKB	Signal transduction pathway that promotes survival and growth in response to extracellular signals. Key proteins involved are PI3K (phosphatidylinositol 3-kinase) and Akt (protein kinase B)
PKC	Protein kinase C
PKR	RNA-activated protein kinase
PPAR	Peroxisome proliferator-activated receptors
PPX	Partial pancreatectomy model
PTHRP	Parathyroid hormone-related protein
RNA	Ribonucleic acid
ROCK	Rho-associated coiled-coil containing protein kinase
S6K1	S6 kinase-1
SAR	Structure-activity relationship
SGLT-2	Sodium-glucose co-transporter-2
SMAD	Family of structurally similar proteins that are the primary signal transducers for receptors of the transforming growth factor-beta
SPRED2	Sprouty-related protein with an EVH1 domain
Sprouty2	Sprouty homolog 2
STAT3	Signal transducer and activator of transcription 3
TGF-β	Transforming growth factor β
TGFβSF	Transforming growth factor-beta superfamily
TNF-α	Tumor necrosis factor α
VGF	Nerve growth factor
